# Förster resonance energy transfer within single chain nanoparticles†

**DOI:** 10.1039/d3sc06651g

**Published:** 2024-02-28

**Authors:** Patrick H. Maag, Florian Feist, Hendrik Frisch, Peter W. Roesky, Christopher Barner-Kowollik

**Affiliations:** a School of Chemistry and Physics, Queensland University of Technology (QUT) 2 George Street QLD 4000 Brisbane Australia; b Centre for Materials Science, Queensland University of Technology (QUT) 2 George Street QLD 4000 Brisbane Australia h.frisch@qut.edu.au christopher.barnerkowollik@qut.edu.au; c Institute of Inorganic Chemistry, Karlsruhe Institute of Technology (KIT) Engesserstraße 15 76131 Karlsruhe Germany roesky@kit.edu; d Institute of Nanotechnology (INT), Karlsruhe Institute of Technology (KIT) Hermann-von-Helmholtz-Platz 1 76344 Eggenstein-Leopoldshafen Germany christopher.barner-kowollik@kit.edu

## Abstract

Single chain nanoparticles (SCNPs) are a highly versatile polymer architecture consisting of single polymer chains that are intramolecularly crosslinked. Currently, SCNPs are discussed as powerful macromolecular architectures for catalysis, delivery and sensors. Herein, we introduce a methodology based on Förster Resonance Energy Transfer (FRET) to evidence the folding of single polymer chains into SCNPs *via* fluorescence readout. We initially introduce a molecular FRET pair based on a bimane and nitrobenzoxadiazole (NBD) moiety and study its fluorescence properties in different solvents. We subsequently construct a low dispersity polymer chain carrying NBD units, while exploiting the bimane units for intramolecular chain collapse. Upon chain collapse and SCNP formation – thus bringing bimane and NBD units into close proximity – the SCNPs report their folded state by a strong and unambiguous FRET fluorescence signal. The herein introduced reporting of the folding state of SCNPs solely relies on an optical readout, opening avenues to monitoring SCNP folding without recourse to complex analytical methodologies.

## Abstract 2

We introduce a methodology based on Förster Resonance Energy Transfer (FRET) to evidence the folding of single polymer chains into single chain nanoparticles (SCNPs) *via* fluorescence readout.

## Graphical abstract

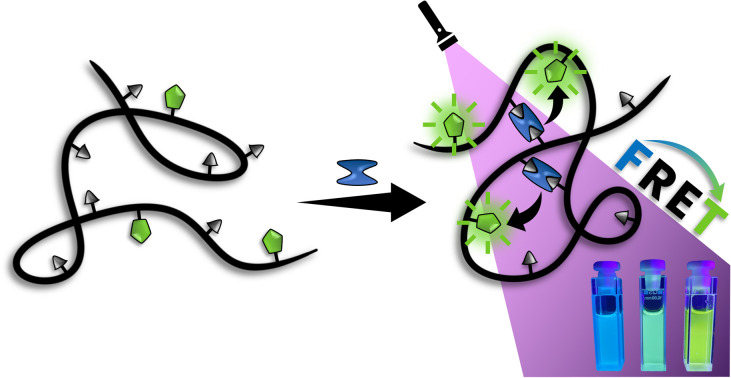

## Introduction

Nature has mastered the folding of (co)polymer chains into complex macromolecular constructs that are information rich and readable.^1^ Polymer chemists have taken inspiration from nature's folding ability and introduced a class of folded synthetic polymer chains today known as single chain nanoparticles (SCNPs), historically also termed nanogels.^2–4^ Over the past decades, SCNPs have been prepared in a wide structural variety *via* a plethora of intramolecular crosslinking chemistries and bonding modes, ranging from covalent to hydrogen bonding, using thermally as well as photochemically driven processes.^5–10^ Modes of folding have been differentiated into repeat unit and selective point folding, which speak to either statistical or directed approaches of intramolecularly connecting the single chain.^11^ Based on these synthetic innovations, SCNPs have been employed as catalysts^12,13^ – fusing the advantages of homo- and heterogeneous catalysis – as well as sensors^14^ and delivery vectors.^15–17^ Concomitantly, the characterization of SCNP morphology and their molecular structure has been elucidated with a wide range of methodologies, including molecular dynamic simulations and microscopic tools as well as NMR spectroscopy and high-resolution mass spectrometry.^16,18^ The perhaps most employed and to date simplest methods for evidencing SCNP compaction are by observing changes in hydrodynamic volume between the unfolded parent polymer and the SCNP *via* dynamic light scattering (DLS)^19–21^ or – more commonly – size exclusion chromatography (SEC).^22,23^ All of the above noted methods, however, require substantial analytical equipment. Elucidating the folding state of SCNPs *via* a simple optical readout would constitute a critical step in establishing – and monitoring – their folding status in a facile manner. Herein, we introduce Förster Resonance Energy Transfer (FRET) to evidence the folding status of SCNPs. FRET is a powerful methodology to gage the distance between molecular entities in a precise fashion, akin to a molecular ruler.^24^ Exploiting the distance-dependence of energy transfer between a donor and acceptor chromophore, changes in proximity of both probes results in an optical response, which has been applied in a variety of different fields.^25–28^ Related to our study, FRET was used to investigate the stability of drug delivery nanogels or micelles and the encapsulation behaviour of fluorophores.^29,30^ FRET has proven to be a powerful tool to study the aggregation behaviour of amphiphiles forming and exchanging between micelles^31^ and was first considered for SCNPs by Stals *et al.* in his PhD thesis.^32^

**Scheme 1:**
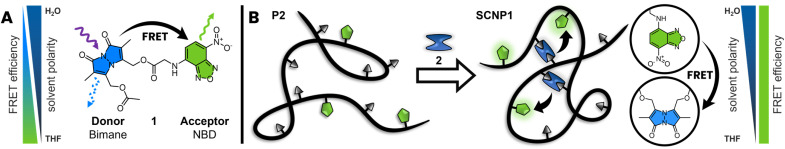
(A) Structure of the molecular FRET pair (1), composed of bimane (blue) and NBD (green), connected with a short linker. Bimane, the energy donor, absorbs light at shorter wavelengths and transfers this energy to the acceptor (NBD), which in turn emits light in its unique spectral range. (B) Incorporating the acceptor into a polymer chain and utilizing 2 as cross-linker allows the optical read out of the folding state *via* the characteristic FRET spectrum.

To utilize FRET to probe SCNP folding, we introduce a new FRET pair, based on the fluorophores bimane and nitrobenzoxadiazole (NBD), combining these into initially one molecule which we carefully assess in a preliminary study (1, refer to Scheme 1A). The choice of bimane unit is driven by its fluorescent properties and commercial availability as difunctionalized dibromobimane (2), able to introduce symmetrical cross-links into the polymer chain. Importantly, the fluorescence of the halogenated bimane species is effectively quenched, making them particularly suitable for use as fluorescent labelling agents.^33–35^ The choice of NBD as counter FRET entity is guided by requiring a moiety that features a comparable fluorescence intensity and shifted emission spectrum compared to the bimane species. We initially demonstrate that the molecular FRET pair 1 functions in organic solvents, specifically tetrahydrofuran (THF), yet shows no FRET activity in aqueous solution. Subsequently, we prepare a water-soluble polymer chain featuring NBD units along its lateral chain as well as carboxylic acid functionalities, which serve as intramolecular crosslinking points. Initiated by dibromobimane 2, the polymer chain is folded into SCNPs, bringing bimane units and NBD together (refer to Scheme 1B). The close proximity and free orientation within SCNPs allow the FRET pair to self-report the folding status by virtue in a variety of solvents, specifically in water. We submit that reading SCNPs' folding status *via* optical means is a unique methodology to not only gain information about their morphological state, but to follow it during, *e.g.*, catalytic applications.

## Results and discussion

To investigate SCNP folding through FRET, we have developed a novel FRET pair composed of the fluorophores bimane and NBD, showing ideal spectral overlap of the donor emission with the acceptor absorbance spectrum. Subsequently, we incorporate both fluorophores in water-soluble SCNPs utilizing dibromobimane 2 as intramolecular crosslinks, facilitating FRET between bimane units and NBD.

### Molecular FRET pair

To study the efficiency and photochemical properties bimane and NBD were combined in a molecular FRET pair (1), utilizing glycine as a short linker. Emission spectra of 1 indicate an efficient energy transfer in THF, yet less efficiency up to no FRET in polar solvents such as water.

**Fig. 1:**
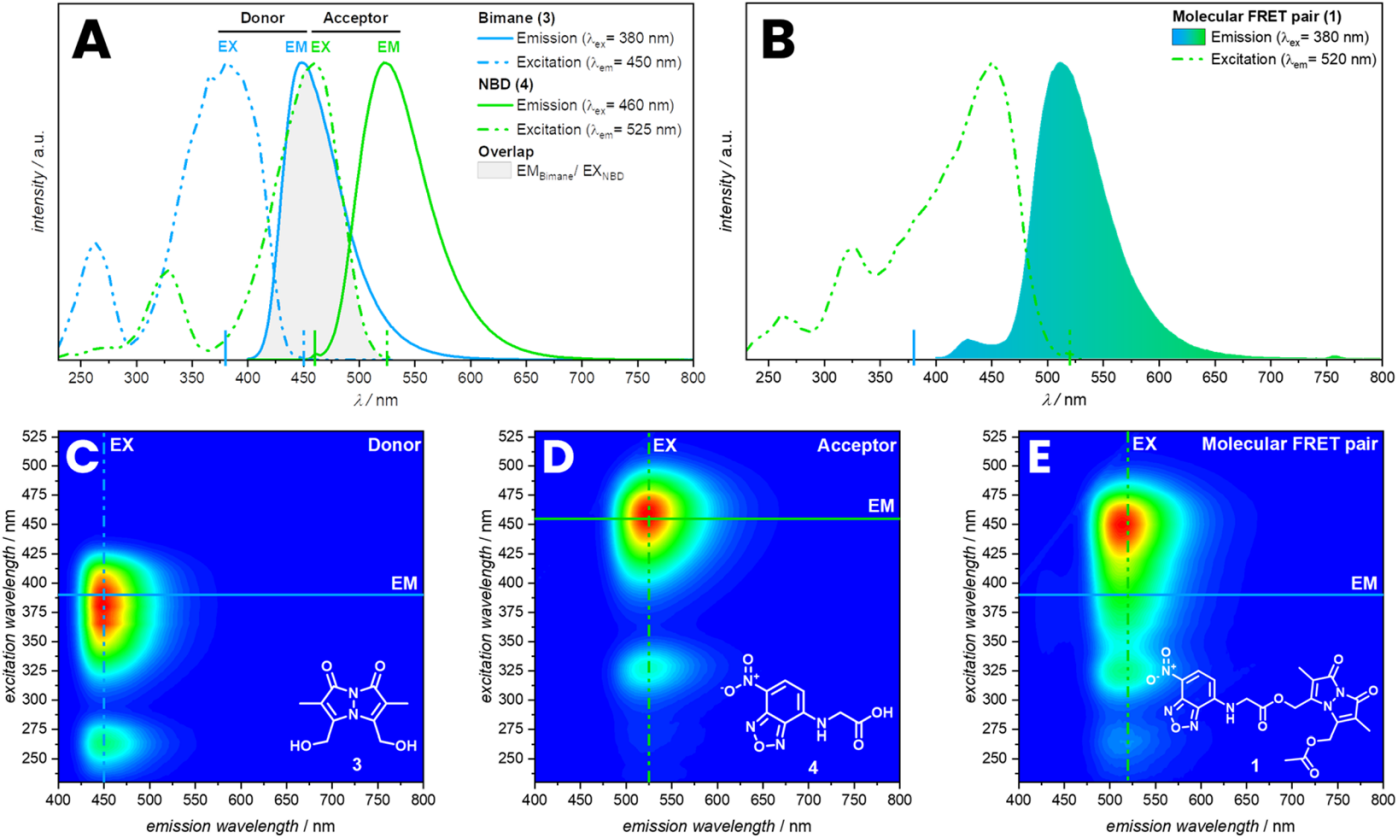
(A) Overlayed fluorescence spectra of 3 and 4 in THF with spectral overlap of the donor emission spectrum with the acceptor excitation spectrum. (B) Fluorescence spectrum of 1 in THF. Bottom: 3D fluorescence spectra of 3 (C), 4 (D) and 1 (E) in THF with indicated 2D slices of the spectra above.

Molecules with a bimane substructure such as dihydroxybimane (3) or the corresponding diacetate (refer to ESI, Section 3.1.2, Fig. S14†) feature fluorescence spectra with a peak emission of 450 nm and an excitation maximum at 380 nm in THF. The fluorescence spectra of molecules with NBD substructure such as (7-nitrobenzo[*c*][1,2,5]oxadiazol-4-yl)glycine (4) are similar to the bimane, yet red-shifted by 80 nm (Fig. 1A). Compound 4 displays a peak emission at 530 nm and the excitation maximum at 460 nm in THF. The local excitation minimum of 4 overlaps with the excitation maximum of 3 and therefore allows for the selective excitation of 3 at 380 nm (Fig. 1A). Of key importance is the spectral overlap of the donor emission with the acceptor absorbance spectrum.^36^ In the present case, the overlap with the acceptor excitation spectrum is identical with the absorbance spectrum (refer to the ESI, Section 3.1.2, Fig. S12†). Compound 1 shows the expected shift in the emission spectrum towards longer wavelengths due to the energy transfer from the donor (bimane) to the acceptor (NBD). Furthermore, the excitation spectrum for an emission of 520 nm combines the excitation spectra of the bimane and the NBD (Fig. 1B). FRET can also be visualized by recording 3D fluorescence spectra of the individual fluorophores and the molecular FRET pair (1). 3 and 4 show their characteristic emission spectrum depending on the excitation wavelength displayed in a heat map (Fig. 1C and D). Combining these into one molecule allows the excitation of both fluorophores and enables the energy transfer to the acceptor, returning the emission spectrum of the NBD (Fig. 1E). The energy transfer can be observed as shift in the intensity to higher emission wavelength.

Fluorescence spectra of 1, 3 and 4 were recorded in THF demonstrate an efficient energy transfer, indicating close proximity and free orientation of both fluorophores. FRET was also observed in DCM, yet was less efficient in acetonitrile and methanol. No FRET was observed in polar solvents as DMF, dimethyl sulfoxide and water (refer to ESI, Section 3, Fig. S5†). The solvent dependent FRET of 1 may be associated with varying orientations of the fluorophores in different solvents. The orientation of emission transition dipole of the donor in comparison to the absorption dipole of the acceptor is an important parameter affecting the Förster distance. The orientation parameter (*κ*^2^) can theoretically range from 0 to 4. However, for a freely rotatable system a value of 2/3 is expected.^36^ Furthermore, earlier studies revealed that solvents effect the rate coefficient of intramolecular FRET and therefore change the efficiency of the energy transfer.^37^ In the case of 1, the solvent might hinder the free orientation of the donor and acceptor or decrease the efficiency based on a change in transfer rate coefficient, yet may also lead to aggregation-induced quenching. Further investigation of this particular FRET pair would require time-resolved spectroscopy.

**Fig. 2:**
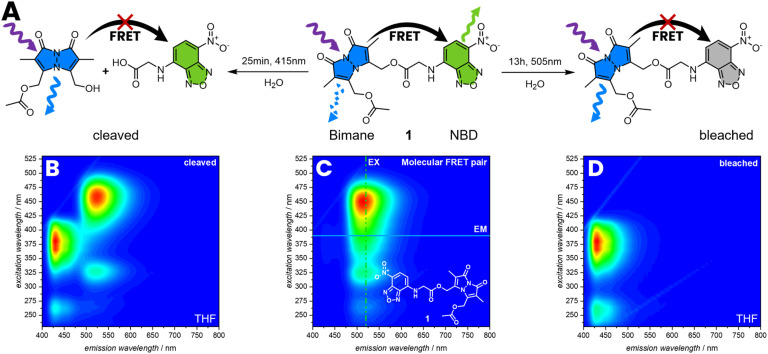
(A) Reaction pathway of cleavage and photobleaching of 1 followed by 3D fluoresce spectroscopy in THF. The cleavage reaction was performed in water for 25 min with *λ*_max_ = 415 nm, resulting in an increasing intensity of the bimane emission (B) compared to (C). To photo-bleach NBD, 1 was irradiated in water for 13 h with *λ*_max_ = 505 nm, resulting in an increasing intensity of the bimane emission (D).

The FRET efficiency was determined by photobleaching of the acceptor, comparing the emission of the bimane before and after bleaching (Fig. 2). Therefore, 1 was irradiated for 13 h with *λ*_max_ = 505 nm until no emissions of the NBD were observed anymore, resulting in an increasing intensity of the bimane emission of 80%. The FRET efficiency is therefore assumed to be close to 80% (refer to the ESI, Section 3.1.2, Fig. S17†). An alternative method to demonstrate the FRET efficiency involves the cleavage of bimane from the NBD. The bimane ester cleavage photoreaction at long irradiation times was studied by Chaudhuri *et al.*^38^ Therefore, 1 was irradiated for 25 min with *λ*_max_ = 415 nm in water resulting in the formation of a carboxylic acid and an alcohol (Fig. 2A and B). The 3D fluorescence spectrum shows the characteristic pattern of both individual fluorophores, indicating that the bond between the donor–acceptor pair is cleaved.

### FRET read-out of SCNP folding

To apply the FRET pair in SCNPs to report their folding state, a water-soluble polymer was synthesized by reversible addition fragmentation chain transfer (RAFT) copolymerization of poly(ethylene glycol) methyl ether methacrylate (MPEGMA, *M*_n_ = 300 g mol^−1^) and acrylic acid (AA). The monomer composition was determined from the conversion of the monomers during polymerization with 16% AA *via*^1^H NMR spectroscopy using DMF as a reference (refer to the ESI, Section 2.2, Fig. S1†). Subsequently, the RAFT end-groups were hydrolysed to form hydroxy groups (refer to the ESI, Section 2.1.2) affording poly(MPEGMA-*co*-AA) (P1, *M*_n_ = 42.6 10^3^ g mol^−1^, *x*_AA_ = 16%).^39^ The molecular weight (*M*_n_) was determined by SEC based on the hydrodynamic volume of the polymer in dimethylacetamide relative to poly(methyl methacrylate) standards.^40^

**Fig. 3:**
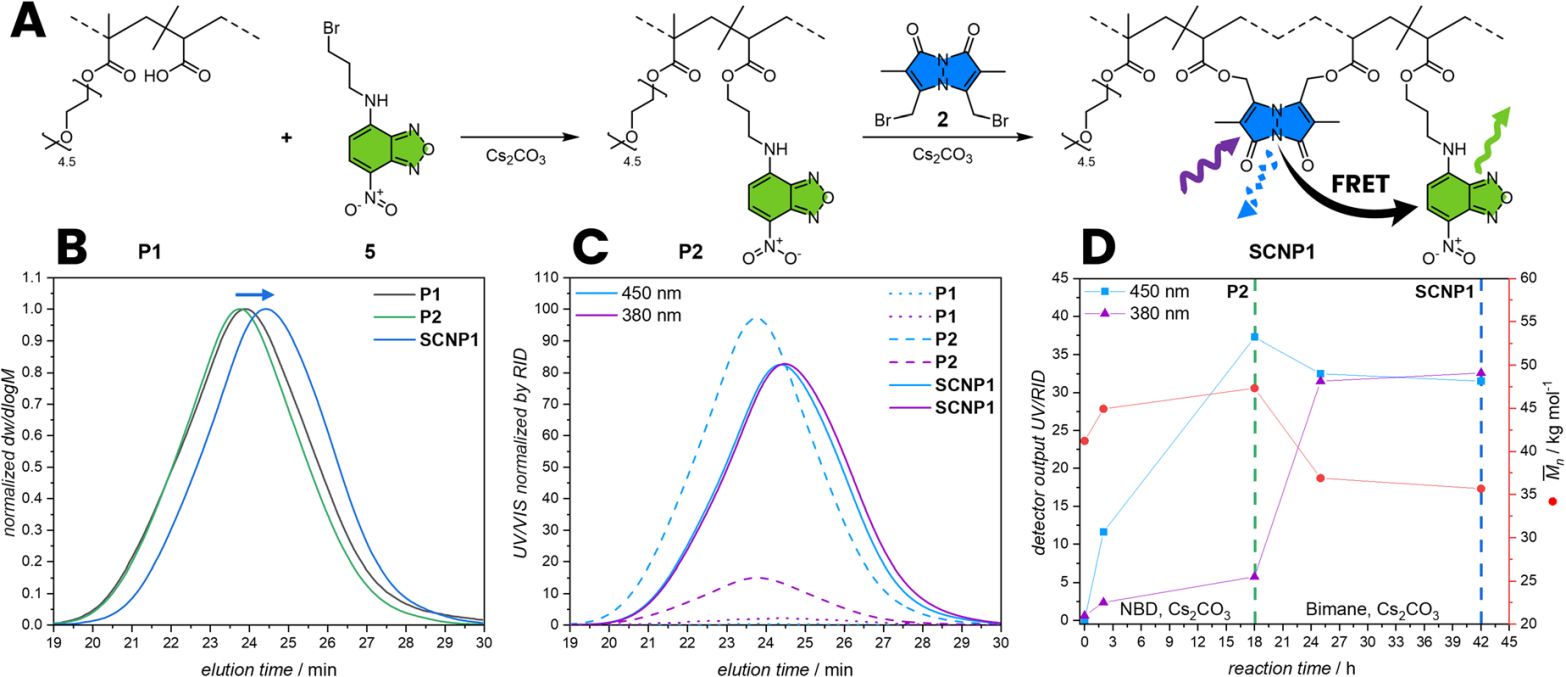
(A) Post-functionalization of P1 with 5 and 2 to incorporating the FRET pair. 2 concomitantly acts as cross-linker, folding P2 into SCNP1. (B) RID SEC traces of P1 post-functionalized to P2 and further folding to SCNP1. (C) UV/Vis absorption SEC trace at 380 nm and 450 nm normalized by RID of P1, P2 and SCNP1. (D) Kinetics of the post-functionalization of P1 to P2 and folding of P2 to SCNP1 monitored by UV/Vis absorption and *M*_n_ by SEC. The cross-linking of the polymer chain leads to a compaction and consequently to a smaller hydrodynamic volume thus to a smaller apparent molecular weight in the SEC. *M*_n_: apparent number average molecular weight.

To functionalise P1 with a FRET acceptor, P1 was post-functionalized with *N*-(3-bromopropyl)-7-nitrobenzo[*c*][1,2,5] oxadiazol-4-amine (5) by an esterification reaction of the carboxylic acid pendant groups in a solution of THF and caesium carbonate as base (P2, *M*_n_ = 47.3 10^3^ g mol^−1^) (Fig. 3A). During esterification with 5, the hydrodynamic volume of the polymer slightly increases, resulting in an increase of the apparent *M*_n_ by 11% (refer to SEC, Fig. 3B) and a slight shift of the P2 SEC elution time compared to the P1. The post-functionalization was closely monitored by comparing the UV/Vis absorption of the polymer in the SEC trace to the refractive index detection (RID). An observed increase in absorption at 450 nm over time evidences the successful incorporation of NBD units within the polymer chain. In a subsequent step, P2 was folded into SCNPs by esterification with dibromobimane 2 in a highly diluted reaction mixture of 0.2 mg mL^−1^P2 in THF (SCNP1, *M*_n_ = 35.7 10^3^ g mol^−1^, Fig. 3D). During folding, the hydrodynamic volume of the polymer decreases, leading to a 25% reduction in the apparent molecular weight (refer to SEC, Fig. 3B), resulting from a shift of the SCNP1 SEC elution time compared to the P2. Moreover, the UV/Vis absorption of the polymer in the SEC trace at 380 nm increases over time, evidencing the successful incorporation of bimane units within the polymer chain, correlating with the decrease in apparent molecular weight and demonstrating that the bimanes act as cross-links. After 2 h of folding, the incorporation rate decreases, reflected by a stagnation of the absorption at 380 nm in the SEC trace, indicating a saturation of the carboxylic acid pendant group.

To investigate if the observed SCNP formation can be monitored through FRET, a 3D fluorescence spectrum of SCNP1 in water was recorded. Pleasingly, it shows a similar pattern as of the directly linked donor–acceptor pair 1 in THF, indicating an efficient FRET between bimane and NBD moieties within the SCNP architecture resulting from a close proximity of crosslink (donor) and the polymer-bound dye (acceptor). To confirm that the observed 3D fluorescence spectrum is the result of FRET, bleaching experiments were carried out (refer to ESI, Section 3.1.4, Fig. S25 and S26†). The efficient energy transfer between the two fluorophores indicates the folded state, which results from their close proximity and the presence of bimane cross-links, whose fluorescence is initially quenched by bromine functions. Herein, we successfully incorporate both fluorophores within SCNPs, paving the way for further quantitative studies to determine the degree of compaction through an optical readout. The presence of FRET in SCNPs in water also supports the notion of a different orientation parameter in different solvents compared to molecule 1 as described above.

**Fig. 4:**
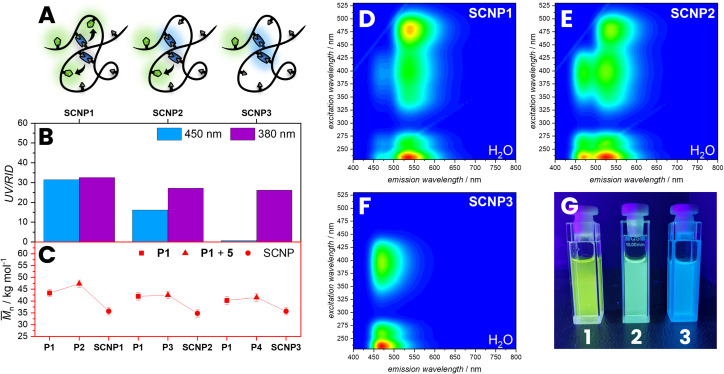
(A) Illustration of SCNPs with different incorporation of NBD (B) analysed by SEC showing different absorption at 450 nm compared to the RID. (C) The apparent *M*_n_ (red) slightly increases after post-functionalization and decreases with folding to SCNPs. (D–F) 3D fluorescence spectra of SCNP1–3 in water. (G) Image of **SCNP1–3** in water irradiated with *λ*_max_ = 380 nm. *M*_n_: apparent number average molecular weight.

Nevertheless, to study the effect of different degrees of NBD incorporation on the FRET efficiency, SCNPs with different amounts of NBD moieties in their polymer backbone were synthesized and 3D fluorescence spectra were recorded. P1 was post-functionalized with different amounts of NBD moieties (5) utilizing the carboxylic acid pendant groups resulting in P3 (*M*_n_ = 42.6 10^3^ g mol^−1^) and P4 (*M*_n_ = 41.6 10^3^ g mol^−1^) with a slightly increased apparent *M*_n_ (Fig. 4). The post-functionalization of NBD units was monitored by UV/Vis absorption at 450 nm of the polymer in the SEC trace showing a decreasing incorporation of NBD moities from P2 to P4. In a subsequent step P3 and P4 were cross-linked with identical amounts of 2 in a highly diluted reaction mixture of 0.2 mg mL^−1^ in THF resulting in SCNP2 (*M*_n_ = 36.1 10^3^ g mol^−1^) and SCNP3 (*M*_n_ = 38.6 10^3^ g mol^−1^). During folding, the hydrodynamic volume of the polymer decreases, leading to a reduction in the apparent molecular weight, resulting from a shift of the SCNP's SEC elution time compared to P3 or P4. With decreasing amount of NBD moieties, it can be observed that the FRET efficiency decreases, observing no FRET in SCNP3. The difference in emission can be visually observed by irradiating with *λ*_max_ = 380 nm LED (Fig. 4G).

**Fig. 5:**
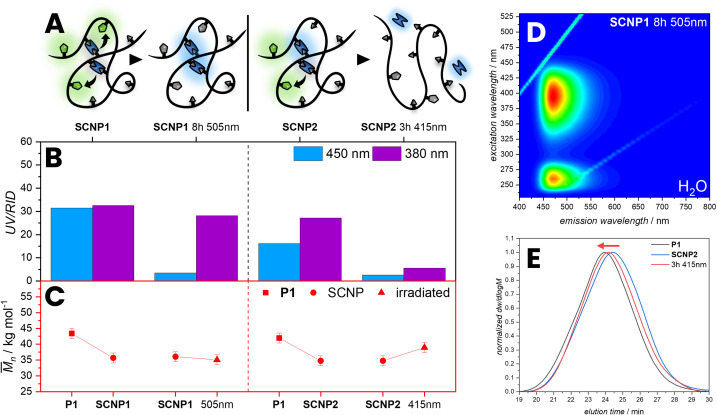
(A) Illustration of SCNP1 irradiated with *λ*_max_ = 505 nm for 8 h resulting in photobleaching of the NBD and SCNP2 irradiated with *λ*_max_ = 415 nm for 3 h resulting in the cleavage and bleaching of the bimane units. (B) SCNP1 and SCNP2 analysed by SEC showing different absorption at 450 nm and 380 nm. (C) The apparent *M*_n_ (red) increases after irradiating with *λ*_max_ = 415 nm in water indicating unfolding of the SCNPs, whereas the apparent *M*_n_ does not increases after irradiating with *λ*_max_ = 505 nm. (D) 3D fluorescence spectrum of SCNP1 irradiated with 505 nm for 8 h in water. (E) RID SEC traces of SCNP2 unfolding compared to P1. *M*_n_: apparent number average molecular weight.

Further, the photochemical behaviour of SCNP1 and SCNP2 was studied. To demonstrate the efficiency of the FRET, SCNP1 was irradiated for 8 h with *λ*_max_ = 505 nm until no emission of the NBD was observed, resulting in an increasing intensity of the bimane emission (Fig. 5). We observed that the *M*_n_ only changes slightly. Additionally, we irradiated the SCNP2 for 3 h with *λ*_max_ = 415 nm in water resulting in a decrease of absorption at 380 nm and 450 nm, but also an increase of apparent molecular weight, suggesting that the SCNPs were unfolded to a linear chain. Irradiating the bimane cross-links within the SCNPs leads to a cleavage of the ester bonds, releasing dihydroxybimane 3 and the original polymer with carboxylic acid pendent groups. The unfolding leads to an increase in the hydrodynamic volume, which concomitantly results in an increase in the apparent molecular weight, which is evidenced by a shift of the irradiated SCNPs SEC elution time compared to the SCNP1 (red arrow, Fig. 5E). The shift of the unfolding in the SEC trace appears not as pronounced as the initial folding, which might be due to a small number of remaining bimane cross-links along the chain or side reactions occurring during the photocleavage and bleaching of NBD moieties. Importantly, such a photochemical unfolding of the SCNP was not observed during the fluorescence measurements, which is perquisite to utilise FRET to probe the SNCP architecture.

## Conclusions

We introduce a molecular FRET pair 1 based on the fluorophores bimane and NBD to study its photochemical properties. The novel FRET pair displays efficient energy transfer between both fluorophores in THF or DCM, changing to no FRET in more polar solvents as DMF and water. Critically, we have successfully developed a methodology using FRET to visualize the folding of single polymer chains into SCNPs. A low dispersity polymer chain carrying NBD units as a FRET acceptor was constructed while exploiting 2 to simultaneously induce intramolecular chain collapse and act as energy donor. SCNPs, upon formation, report their folded state through a strong FRET fluorescence signal. The presented optical readout method for reporting the folded state of SCNPs is paving the way for *in situ* monitoring of SCNP folding, circumventing the need for complex analytical methodologies.

## Data availability

Experimental data is available in the ESI.† Additional data is available upon request from the corresponding authors.

## Author contributions

P. H. M. conducted all experimental work, supported by F. F., C. B.-K., P. W. R. and H. F. motivated and supervised the study. C. B.-K. and P. W. R. acquired funding.

## Conflicts of interest

There are no conflicts to declare.

## Supplementary Material

SC-015-D3SC06651G-s001
