# Supplementary material for: Förster resonance energy transfer within single chain nanoparticles
Source: Chem Sci. 2024 Feb 28;15(14):5218–24. doi: 10.1039/d3sc06651g (PMC10988607; doi:10.1039/d3sc06651g)
Supplement: SC-015-D3SC06651G-s001 [file SC-015-D3SC06651G-s001.pdf]

Electronic Supplementary Material (ESI) for Chemical Science.
This journal is © The Royal Society of Chemistry 2024

Electronic Supporting Information

Förster Resonance Energy Transfer within Single Chain Nanoparticles

Patrick H. Maag,[a,b,c] Florian Feist,[c] Hendrik Frisch,*[a] Peter W. Roesky,*[b] Christopher Barner-Kowollik*[ac]

Table of Contents

1. Experimental Details 3
  1.1. Materials 3
  1.2. DMAC-Size Exclusion Chromatography (DMAc-SEC) 3
  1.3. Liquid Chromatography – Mass Spectrometry (LC-MS) 3
  1.4. 1D NMR Measurements 3
  1.5. UV-VIS Spectroscopy 3
  1.6. Fluorescence Spectroscopy 4
  1.7. Puriflash Liquid Chromatography 4
2. Experimental Procedures 5
  2.1. Polymer synthesis/ post-functionalization 5
  2.1.1. RAFT co-polymerization to form poly(poly(ethylene glycol) methyl ether methacrylate-co-acrylic acid) (poly(MPEGMA-co-AA)) 5
  2.1.2. End group modification of poly(poly(ethylene glycol) methyl ether methacrylate-*co*-acrylic acid) yielding P1 5
  2.1.3. Post-functionalization and SCNPs formation (SCNP1) 5
  2.1.4. Post-functionalization and SCNPs formation (SCNP2) 6
  2.1.5. Post-functionalization and SCNPs formation (SCNP3) 6
  2.2. Analysis of Copolymer Composition 6
  2.3. Small Molecule Synthesis 7
  2.3.1. *tert*-Butyl (7-nitrobenzo[c][1,2,5]oxadiazol-4-yl)glycinate (NBD-Gly-*tert*) 7
  2.3.2. (7-Nitrobenzo[c][1,2,5]oxadiazol-4-yl)glycine (**4**) 8
  2.3.3. (5-(Acetoxymethyl)-2,6-dimethyl-1,7-dioxo-1H,7H-pyrazolo[1,2-a]pyrazol-3-yl)methyl (7-nitrobenzo[c][1,2,5]oxadiazol-4-yl)glycinate (**1**) 8
  2.3.4. *N*-(3-bromopropyl)-7-nitrobenzo[c][1,2,5] oxadiazol-4-amine (**5**) 8
  2.3.5. Dihydroxybimane (**3**) 9
  2.4. Irradiation Experiments 9
  2.4.1. Irradiation experiment of 1 10
  2.4.2. Unfolding of SCNP2 by radiation with 415 nm 10
3. Fluorescence spectra 11
  3.1.1. Fluorophores in H_2O 12
  3.1.2. Fluorophores in THF 13
  3.1.3. Irradiation Experiments 16
  3.1.4. Polymers 17
4. LCMS 22
5. NMR Spectra 25
  5.1. Polymer 25
  5.2. Small Molecule 27
6. References 31

1. Experimental Details

1.1. Materials

All chemicals were used as received unless noted otherwise. 2-cyano-2-propyl benzodithioate (Sigma-Aldrich, >97%), 4-chloro-7-nitrobenzofurazan (Sigma-Aldrich, 98%), acetic acid (Fisher chemical, >99.0%), acetonitrile (**ACN**, Fisher chemical, >99.9%), acrylic acid (**AA**, anhydrous, Sigma-Aldrich, contains 200 ppm MEHQ as inhibitor, 99%), azobisisobutyronitrile (**AIBN**, Sigma-Aldrich, 12.-wt% in acetone), cesium carbonate (Alfa Aesar, 99.5%), Cyclohexane (Sigma-Aldrich, EMSURE®), dibromobimane (**2**, Synchem, 98%), dichloromethane (fischer scientific, >99.8%) diethyl ether (Ajax Finechem, >99.5), dimethyl sulphoxide (Ajax Finechem, >99.0), ethanol (Ajax Finechem, absolute, >99.5%), ethyl acetate (Fisher chemical, ≥99.8%), glycine *tert*-butyl ester HCl (Combi-Blocks, 98%), magnesium sulfate anhydrous (Sigma-Aldrich), methanol (Ajax Finechem, >99.9), N,N-Dimethylformamide (**DMF**, Sigma-Aldrich, 99.8%), n-pentane (Ajax Finechem, absolute, >98%), poly(ethylene glycol) methyl ether methacrylate (average Mn 300, **MPEGMA**, Sigma-Aldrich, contains 100 ppm MEHQ as inhibitor, 300 ppm BHT as inhibitor), sodium formate (Sigma-Aldrich), sodium hydrogen carbonate (Ajax Finechem, absolute, >99.7%), triethylsilane (Sigma-Aldrich, >99%), tetrahydrofuran (**THF**, Fisher chemical, >99.9%), trifluoroacetic acid (thermos scientific, 99%), triphenylphosphine (ChemSupply, >99%).

1.2. DMAC-Size Exclusion Chromatography (DMAc-SEC)

DMAc-SEC measurements were conducted on a *PSS* SECurity2 system consisting of a *PSS* SECurity Degasser, *PSS* SECurity TCC6000 Column Oven (60 °C), PSS GRAM Column Set (8x150 mm 10 µm Precolumn, 8x300 mm 10 µm Analytical Columns, 1000 Å, 1000 Å and 30 Å) and an Agilent 1260 Infinity Isocratic Pump, *Agilent* 1260 Infinity Standard Autosampler, *Agilent* 1260 Infinity Diode Array and Multiple Wavelength Detector (A: 380 nm, B: 450 nm), *Agilent* 1260 Infinity Refractive Index Detector (RID, 35 °C). HPLC grade DMAc, 0.01 M LiBr, is used as eluent at a flow rate of 1 mL min^{-1}. Narrow disperse linear poly(methyl methacrylate) (PMMA, M_n: 202 g mol^{-1} to $2.2x10^6$ g mol^{-1}) standard (*PSS* ReadyCal) was used as calibrant. All samples were passed over 0.22 µm PTFE membrane filters. Molecular weight and dispersity analysis were performed in *PSS* WinGPC UniChrom software (version 8.2).

1.3. Liquid Chromatography – Mass Spectrometry (LC-MS)

LC-MS measurements were performed on an *UltiMate* 3000 UHPLC System (*Dionex*) consisting of a pump (LPG 3400SZ), autosampler (WPS 3000TSL), and a temperature-controlled column compartment (TCC 3000). Separation was performed on a C18 HPLC column (*Phenomenex* Luna 5µm, 100 Å, 250 × 2.0 mm) operating at 40 °C. Water (containing 5 mmol L^{-1} ammonium acetate) and acetonitrile were used as eluents. A gradient of acetonitrile: H_2O, 5:95 to 100:0 (v/v) in 7 min at a flow rate of 0.40 mL·min^{-1} was applied. The flow was split in a 9:1 ratio, where 90% of the eluent was directed through a DAD UV-detector (VWD 3400, *Dionex*) and 10% was infused into the electrospray source. Spectra were recorded on an LTQ Orbitrap Elite mass spectrometer (*Thermo Fisher Scientific*) equipped with a HESI II probe. The instrument was calibrated in the *m*/*z* range 74-1822 using premixed calibration solutions (*Thermo Scientific*). A constant spray voltage of 3.5 kV, a dimensionless sheath gas, and a dimensionless auxiliary gas flow rate of 5 and 2 were applied, respectively. The capillary temperature was set to 300 °C, the S-lens RF level was set to 68, and the aux gas heater temperature was set to 100 °C.

1.4. 1D NMR Measurements

^{1}H and ^{13}C MR spectra were recorded on a *Bruker* Avance III 600 MHz, equipped with a BBO-Probe (5 mm) with z-gradient (^{1}H: 600.13 MHz, ^{13}C: 150.90 MHz,) or a *Bruker* Avance III 400 MHz spectrometer equipped with a Quattro Nucleus Probe (QNP) with an operating frequency of 400 MHz (^{1}H). All measurements were carried out in deuterated solvents. The chemical shift (δ) is recorded in parts per million (ppm) and relative to the residual solvent protons.[1] The measured coupling constants were calculated in Hertz (Hz). Spectra were analyzed with *MestreLab* MNova 11.0. The signals were quoted as follows: s = singlet, d = doublet, t = triplet, and m = multiplet.

1.5. UV-VIS Spectroscopy

UV/vis spectra were recorded on a *Shimadzu* UV-2700 spectrophotometer equipped with a CPS-100 electronic temperature control cell positioner. Samples were dissolved, filtered, and subsequently measured in *Hellma Analytics* quartz high precision cells with a path length of 10 mm at ambient temperature.

1.6. Fluorescence Spectroscopy

The fluorescence spectra were recorded on a Cary Eclipse Fluorescence Spectrophotometer from *Agilent Technologies*. Voltage was adjusted between 400 V and 700 V with an excitation and emission slit of 5 nm (scan rate 1200 nm min^{-1}, data interval 2 nm). Samples were measured at ambient temperature in *Hellma Analytics* quartz high precision cells with a path length (l) of 10 mm. 3D spectra were recorded in the emission scan mode with an excitation wavelength of 230 nm to 530 nm with increments of 5 nm. To ensure a linear behavior of the emission intensity as a function of concentration, the absorption of the solution was adjusted to 0.1 %.

1.7. Puriflash Liquid Chromatography

Flash chromatography was performed on an *Interchim* XS420Plus flash chromatography system consisting of an SP-in-line filter (20 µm), and a UV-VIS detector (200-800 nm). The separations were performed using *Interchim* dry load columns (compound adsorbed on celite for dry loading, wet loading via syringe) and *Interchim* Puriflash Silica HP 30 µm. Flow rate is typically 25 mL min^{-1} resulting in < 1 bar pressure.

2. Experimental Procedures

2.1. Polymer synthesis/ post-functionalization

2.1.1. RAFT co-polymerization to form poly(poly(ethylene glycol) methyl ether methacrylate-co-acrylic acid) (poly(MPEGMA-co-AA))

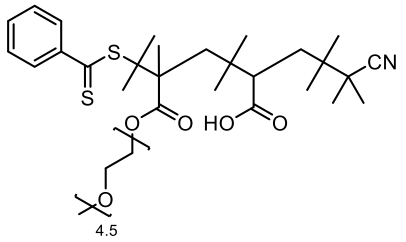

The procedure was adapted from Irshadeen *et al.*[2] Poly(ethylene glycol) methyl ether methacrylate and acrylic acid was passed through a short neutral alumina column to remove the inhibitor before being utilized in the polymerization. Poly(ethylene glycol) methyl ether methacrylate (M_n = 300 g mol^{-1}, 2.79 mL, 300.00 eq, 13.52 mmol), acrylic acid (309.00 µL, 100.00 eq, 4.51 mmol), 2-cyano-2-propyl benzodithioate (9.97 mg, 1.00 eq, 45.06 µmol) and AIBN (12 wt. % in acetone, 9.97 µL, 0.20 eq, 9.01 µmol) were added to a 10 mL Schlenk tube and dissolved in 10 mL of DMF. The reaction mixture was degassed by passing through a stream of N_2 for 10 min. The mixture was subsequently heated at 70 °C for 22 h. The polymer was precipitated by dropwise addition of the crude reaction mixture to cold Et_2O/pentane (5:1) and isolated via centrifugation. The polymer was redissolved in THF and the precipitation process was repeated. Afterwards the polymer was dried under high vacuum.

SEC characterization (THF, RI): M_n = 47.24 10^3 g mol^{-1}, $Đ$ = 1.37.

2.1.2. End group modification of poly(poly(ethylene glycol) methyl ether methacrylate-*co*-acrylic acid) yielding P1

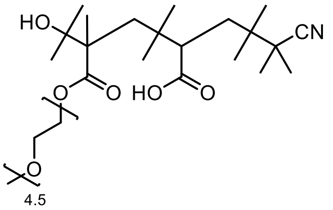

The procedure was adapted from Dietrich *et al.*[3] A solution of 2,2′-azobis(isobutyronitrile) (14.79 mg, 0.09 mmol, 10 mmol L^{-1}) in 9 mL of THF was heated at 60 °C for 60 min under ambient atmosphere. A solution of 3.00 g RAFT-polymer in the pre-treated THF (10 mmol L^{-1} based on M_n) was prepared in a 50 mL round flask under ambient atmosphere. The flask was subsequently heated to 60 °C under vigorous stirring. After the discoloration of the solution indicated full conversion of the RAFT end group, the temperature was reduced to 40 °C and 3.00 eq triphenylphosphine were added. After 15 min the polymer was precipitated by dropwise addition of the crude reaction mixture to cold Et_2O/pentane (5:1) and isolated via centrifugation. The polymer was redissolved in THF and the precipitation process was repeated. Afterwards the polymer was dried under high vacuum.

SEC characterization (DMAc, RI): M_n = 42.62 10^3 g mol^{-1}, $Đ$ = 1.62.
^{1}H NMR (400 MHz, $CDCl_3$) δ 4.43 – 3.92 (m), 3.75 – 3.60 (m), 3.59 – 3.53 (m), 3.39 (bs), 2.25 – 1.68 (m), 1.21 – 1.08 (m), 1.09 – 0.94 (m), 0.94 – 0.82 (m).

2.1.3. Post-functionalization and SCNPs formation (SCNP1)

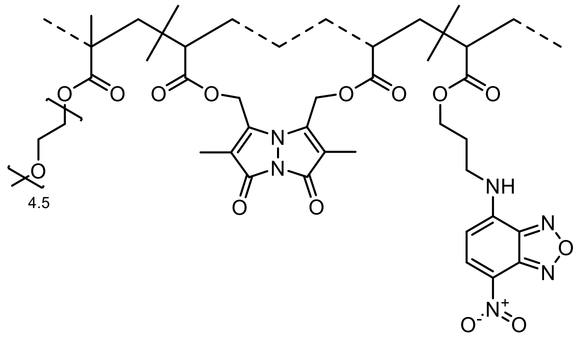

P1 (50.00 mg, equivalent to 30.74 µmol AA units, 1.00 eq) and cesium carbonate (5.01 mg, 15.37 µmol, 0.50 eq) was dissolved in 2.5 mL of THF in a 500 mL round bottom flask (brown glass). **5** (4.6 mg, 15.37 µmol, 0.50 eq) was added and stirred for 18 h.

Subsequently, the reaction mixture was diluted with 250 mL THF and dibromobimane **2** (5.01 mg, 30.74 µmol, 0.50 eq) was added and stirred further 24 h. Next, the solvent was evaporated, and the polymer was purified by dialysis (cellulose membrane, 10 kDa MWCO) in methanol affording **SCNP1**, which was stored in a solution of MeOH (10 mg mL) to prevent side reaction and precipitation.

SEC characterization (DMAc, RI): M_n = 35.67 10^3 g mol^{-1}, $Đ$ = 1.57.
^{1}H NMR (600 MHz, MeOD) δ 8.61 (s), 6.50 (s), 5.45 (bs), 4.13 (bs), 3.66 (bs), 3.56 (bs), 3.38 (bs), 2.30 – 1.66 (m), 1.25 – 0.79 (m).

2.1.4. Post-functionalization and SCNPs formation (SCNP2)

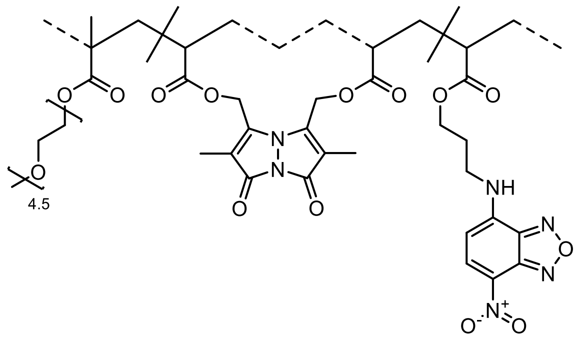

P1 (50.00 mg, equivalent to 30.74 µmol AA units, 1.00 eq) and cesium carbonate (5.01 mg, 15.37 µmol, 0.50 eq) was dissolved in 2.5 mL of THF in a 500 mL round bottom flask (brown glass). **5** (4.6 mg, 15.37 µmol, 0.50 eq) was added and stirred for 2 h. Subsequently, the reaction mixture was diluted with 250 mL of THF and dibromobimane **2** (5.01 mg, 30.74 µmol, 0.50 eq) was added and stirred further 3 h. Next, the solvent was evaporated and the polymer was purified by dialysis (cellulose membrane, 10 kDa MWCO) in methanol affording **SCNP2** which was stored in a solution of MeOH (10 mg mL) to prevent side reaction and precipitation.

SEC characterization (DMAc, RI): M_n = 36.13 10^3 g mol^{-1}, $Đ$ = 1.65.
^{1}H NMR (600 MHz, MeOD) δ 8.63 (s), 6.51 (s), 5.37 (bs), 4.13 (bs), 3.86 – 3.47 (m), 3.38 (bs), 2.34 – 1.41 (m), 1.22 – 0.72 (m).

2.1.5. Post-functionalization and SCNPs formation (SCNP3)

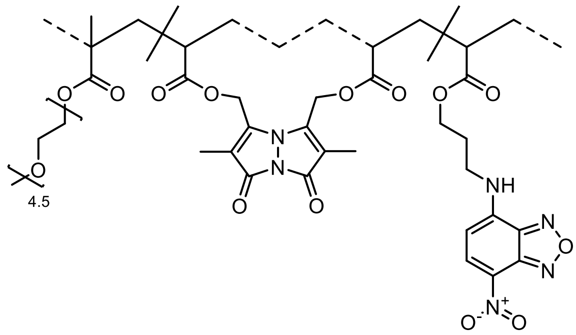

P1 (50.00 mg, equivalent to 30.74 µmol AA units, 1.00 eq) and cesium carbonate (5.01 mg, 15.37 µmol, 0.50 eq) was dissolved in 2.5 mL of THF in a 500 mL round bottom flask (brown glass). **5** (0.93 mg, 3.07 µmol, 0.10 eq) was added and stirred for 2 h. Subsequently, the reaction mixture was diluted with 250 mL THF and dibromobimane **2** (5.01 mg, 30.74 µmol, 0.50 eq) was added and stirred further for 3 h. Next, the solvent was evaporated and the polymer was purified by dialysis (cellulose membrane, 10 kDa MWCO) in methanol, affording **SCNP3** which was stored in a solution of MeOH (10 mg mL) to prevent side reaction and precipitation.

SEC characterization (DMAc, RI): M_n = 38.62 10^3 g mol^{-1}, $Đ$ = 1.62.
^{1}H NMR (600 MHz, MeOD) δ 5.62 – 5.08 (m), 4.13 (bs), 3.91 – 3.46 (m), 3.38 (bs), 2.43 – 1.42 (m), 1.41 – 0.83 (m).

2.2. Analysis of Copolymer Composition

The monomer composition within the polymer was determined by the monomer conversion during polymerization monitored by ^{1}H NMR spectroscopy in deuterated chloroform. As internal standard, the resonance of the solvent DMF ($(CH_3)_2NC(O)$**H**, 7.62 – 8.59 ppm) was used, resulting in conversion 79 mol% poly(ethylene glycol) methyl ether methacrylate (MPEGMA) and 46 mol% acrylic acid (AA). Considering the reactant composition from **2.1.1**, the poly(MPEGMA-*co*-AA) contains 84 mol% MPEGMA units and 16 mol% AA.

Table 1: Analysis of copolymer composition by conversion of the polymerization.

Monomer	Group	^{1}H ∫ *-Area* ppm	*Conversion* %	*Eq.*	*Composition* %
MPEGMA	CH_3C=**CH_2**	5.45 – 5.57	78.6	300	84
AA	HC=**CH_2**	5.72 – 5.82	45.5	100	16

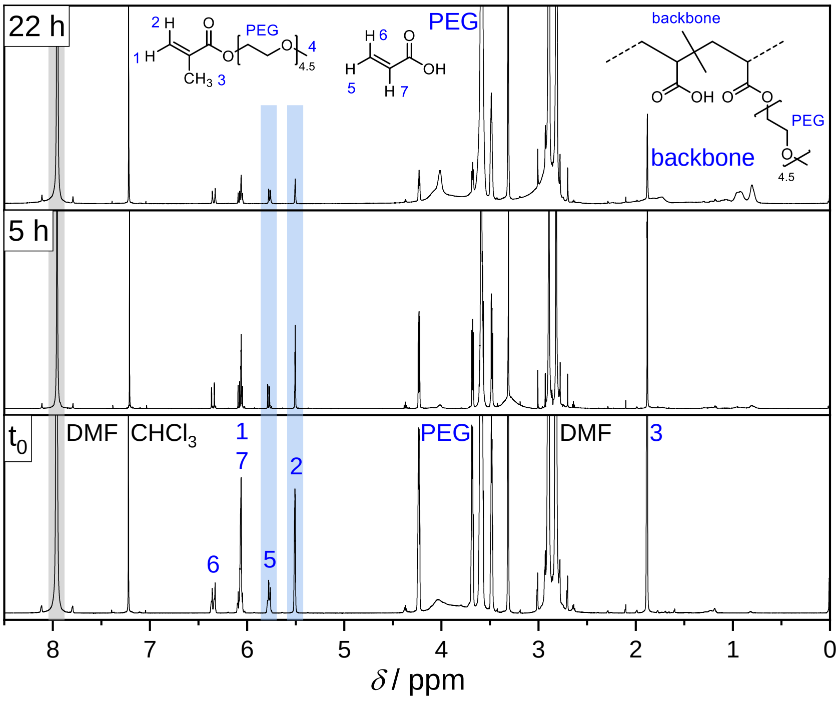

Figure S1: ^{1}H NMR spectrum of the *co*-polymerization after 5 and 22 h. The last one was used for the calculation of the copolymer composition.

2.3. Small Molecule Synthesis

2.3.1. *tert*-Butyl (7-nitrobenzo[c][1,2,5]oxadiazol-4-yl)glycinate (NBD-Gly-*tert*)

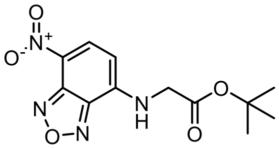

The procedure was adapted from Greco *et al.*[4] Glycine *tert*-butyl ester (1.68 g, 5.01 mmol, 2.00 eq), 4-chloro-7-nitrobenzo-2-oxa-1,3-diaziole (0.51 g, 2.51 mmol, 1.00 eq) and $NaHCO_3$ (0.63 g, 7.52 mmol, 3.00 eq) were dissolved in 25 mL of methanol. The mixture was stirred under light exclusion at room temperature. After 16 h, the solvent was evaporated and the residue dissolved in 100 mL of ethyl acetate, the organic phase was washed with 3x 50 mL of 1 N HCl, 25 brine, dried over $MgSO_4$ and filtered. The solvent was evaporated. The crude product was purified by column chromatography on silica gel (gradient CH:EE 70:30-20:80 *v/v*) to afford the product as an orange powder.

^{1}H NMR (600 MHz, CD_3CN) δ 8.50 (d, J = 8.7 Hz, 1H), 7.28 (s, 1H), 6.28 – 6.24 (m, 1H), 4.20 (s, 2H), 1.47 (s, 9H).
^{13}C NMR (151 MHz, CD_3CN) δ 168.5, 145.7, 145.3, 137.9, 125.0, 100.8, 83.5, 46.4, 28.2.

2.3.2. (7-Nitrobenzo[c][1,2,5]oxadiazol-4-yl)glycine (4)

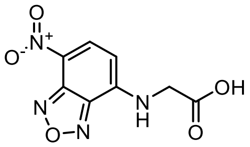

tert-Butyl (7-nitrobenzo[c][1,2,5]oxadiazol-4-yl)glycinate (50.00 mg, 0.17 mmol, 1.00 eq) was dissolved in 1 mL of trifluoroacetic acid and 0.1 mL triethylsilane was added. After 90 min, the reaction mixture was dissolved in 50 mL ethyl acetate, the organic phase was washed with 3x 50 mL of 1 N HCl, 25 brine, dried over $MgSO_4$ and filtered. The product was afforded as an orange powder.

^{1}H NMR (600 MHz, MeOD) δ 8.52 (d, J = 8.7 Hz, 1H), 6.30 (s, 1H), 4.32 (s, 2H).
^{13}C NMR (151 MHz, MeOD) δ 171.8, 145.9, 145.4, 138.0, 127.8, 124.5, 100.6, 45.4.

2.3.3. (5-(Acetoxymethyl)-2,6-dimethyl-1,7-dioxo-1H,7H-pyrazolo[1,2-a]pyrazol-3-yl)methyl (7-nitrobenzo[c][1,2,5]oxadiazol-4-yl)glycinate (1)

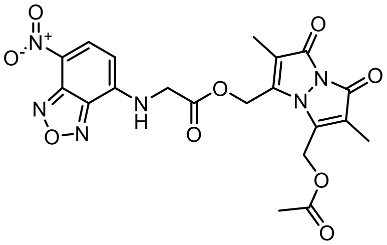

4 (10.00 mg, 0.04 mmol, 1.00 eq), dibromobimane (22.00 mg, 0.04 mmol, 1.50 eq), and Cs_2CO_3 (6.84 mg, 0.02 mmol, 0.50 eq) were dissolved in 5 mL of THF and stirred at ambient temperature for 6 h. Cs_2CO_3 (123 mg, 0.38 mmol, 9.00 eq) and acetic acid (0.024 mL, 0.42 mmol, 10.00 eq) were added and stirred for additional 2 h. The reaction mixture was dissolved in 50 mL of ethyl acetate, the organic phase was washed with 3x 50 mL of 1 N HCl, 25 brine, dried over $MgSO_4$ and filtered. The crude product was purified by column chromatography on silica gel (gradient DCM:MeOH 98:2-92:8 *v/v*) to afford the product as an orange powder.

^{1}H NMR (600 MHz, CD_3CN) δ 8.48 (d, J = 8.6 Hz, 1H), 6.29 (d, J = 6.5 Hz, 1H), 5.24 (s, 2H), 5.01 (s, 2H), 4.40 (s, 2H), 2.05 (s, 3H), 1.88 (s, 3H), 1.83 (s, 3H).
^{13}C NMR (151 MHz, CD_3CN) δ 170.8, 161.0, 144.0, 143.3, 137.5, 116.5, 56.4, 55.5, 20.7, 7.2, 7.1.

2.3.4. *N*-(3-bromopropyl)-7-nitrobenzo[c][1,2,5] oxadiazol-4-amine (5)

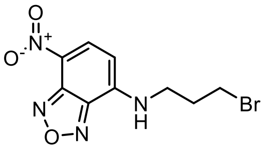

The procedure was adapted from Buscher *et al*.[5] 4-Chloro-7-nitrobenzo[c][1,2,5]oxadiazole (100 mg, 0.50 mmol, 1.00 eq.) was dissolved in EtOAc (3.0 mL). 3-Bromopropylamine (109.70 mg, 0.50 mmol, 1.00 eq.) and $NaHCO_3$ (126.26 mg, 1.50 mmol, 3.00 eq.) were added and the suspension was stirred at 50 °C for 3 d. The reaction mixture was diluted with 50 mL of ethyl acetate, the organic phase was washed with 3x 50 mL 1 N HCl, 25 brine, dried over $MgSO_4$ and filtered. The crude product was purified by column chromatography on silica gel (gradient CH:EE 75:25-45:55 *v/v*) to afford the product as an orange powder (63 mg).

^{1}H NMR (600 MHz, CD_3CN) δ 8.49 (d, J = 8.8 Hz, 1H), 7.39 (s, 1H), 6.34 (d, J = 8.8 Hz, 1H), 3.66 (s, 2H), 3.58 (t, J = 6.5 Hz, 2H), 2.27 (p, J = 6.6 Hz, 2H).
^{13}C NMR (151 MHz, CD_3CN) δ 144.4, 145.3, 144.9, 137.8, 123.5, 99.5, 42.5, 31.5, 31.4.

2.3.5. Dihydroxybimane (3)

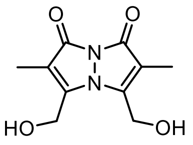

Dibrombimane (10.00 mg, 0.03 mmol, 1.00 eq) was dissolved in ethanol and sodium formate (19.40 mg, 0.29 mmol, 10.00 eq) was added. The reaction mixture was heated overnight at 80 °C, cooled at ambient temperature, filtered and the solvent evaporated to obtain dihydroxybimane **3** without further purification. The product was characterized by LC-MS (Figure S31).

2.4. Irradiation Experiments

The photochemical reactions were performed using a 10 mL crimp cap vial or a 250 mL round-bottom flask and LED (10 W Violet LED, λ_{max} = 415 nm or 505 nm, EPILED from *Future Eden Ltd.*). The LEDs were powered by a 10 W power supply and positioned in a distance of 5 cm measured from the center of the vials (Figure S2). The LEDs and vials were cooled by a small ventilator to maintain ambient temperature of 20 to 24 °C. The aqueous solution was degassed by passing through a stream of nitrogen for at least 10 min. Individual procedures are described at Chapter 2.4.2.

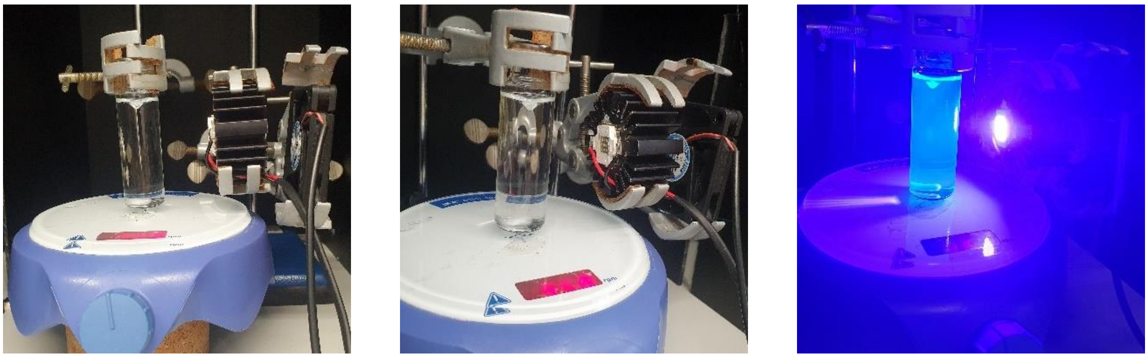

Figure S2: Irradiation setup for the photo cleavage of bimane cross-links with visible light. The sample was mixed with water, degassed by passing a stream of N_2 through the solution for 10 min, sealed and stirred with an electric stir bar. The LED and reaction mixture were cooled by a small electric fan to maintain ambient temperature. All three pictures show the same setup from different angels without and under irradiation.

LED emission spectra were recorded using an Ocean Insight Flame-T-UV-Vis spectrometer, with an active range of 200-850 nm and an integration time of 10 ms. The emission spectra of the employed LEDs are depicted in Figure S3.

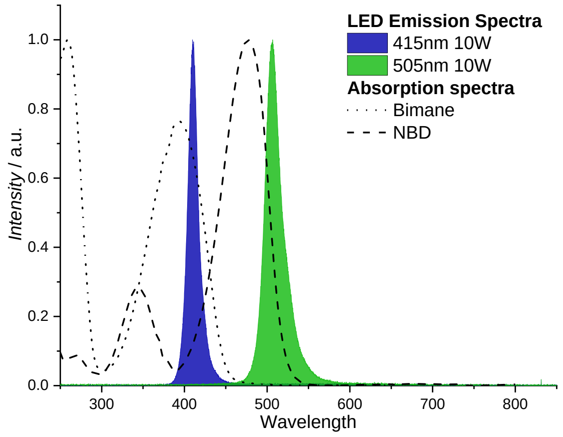

Figure S3: Emission spectrum of 415 nm (green) and 505 nm (blue) 10 W LEDs and absorbance spectrum of bimane and NBD (black).

2.4.1. Irradiation experiment of 1

Compound **1** (30 µL stock solution, 5 mg/mL) was diluted in 5 mL water and degassed by passing through a stream of nitrogen for 10 min using a 10 mL crimp cap vial. The solution was irradiated with 415 nm for 25 min. The reaction mixture was analyzed by fluorescence spectroscopy and LCMS. The photoreaction products were analyzed by LCMS are shown in Figure S4.

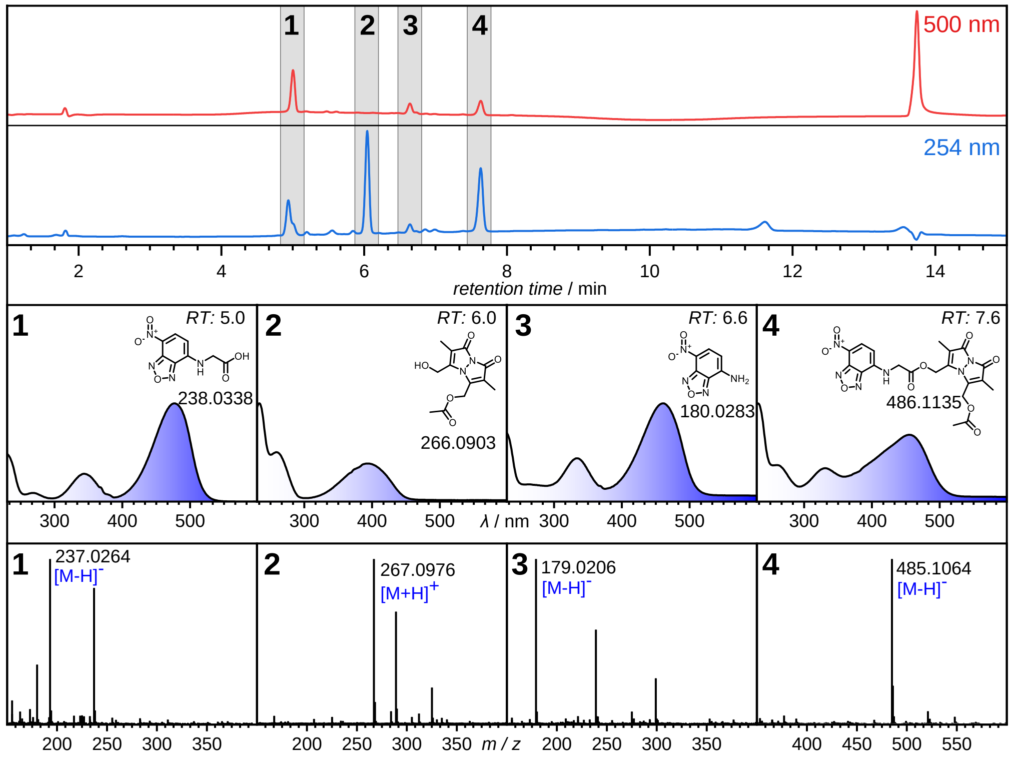

Figure S4: LC-MS trace (254 nm, 500 nm detector wavelength), absorption spectra and accumulated mass spectrum of **1** irradiated with 415 nm for 25 min.

2.4.2. Unfolding of SCNP2 by radiation with 415 nm

A solution of **SCNP2** (2.00 mg, in 300 µL MeOH) was dissolved in 10 mL H_2O (0.2 mg mL^{-1}) and degassed by passing a stream of N_2 through the solution for 10 min. The solution was subsequently irradiated for 3 h and analyzed by fluorescence spectroscopy and SEC. The SEC sample was prepared adding 0.5 mL DMAc to the reaction mixture and evaporating the water under reduced pressure.

SEC characterization (DMAc, RI): M_n = 38.95 10^3 g mol^{-1}, $Đ$ = 1.59.

3. Fluorescence spectra

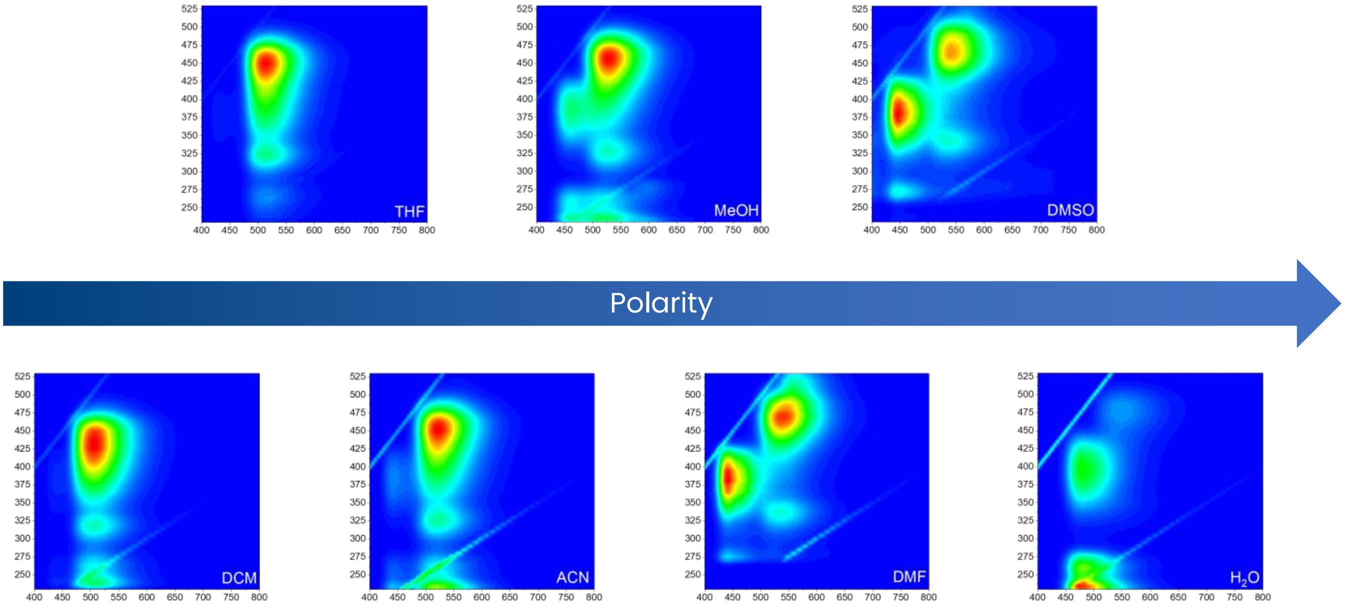

Figure S5: 3D Fluorescence spectra of **1** in different solvents sorted by polarity. Less polar solvents show a more efficient energy transfer between donor (bimane) and acceptor (NBD) then polar solvents.

Absorption at 475 nm of **1** was compared to the emission intensity at 550 nm. Figure S6 shows a linear behavior of the absorption at different concentration in water, however the emission intensity is only linear until 0.2 % absorption. To ensure a linear behavior of the emission intensity compared to the concentration the absorption of the solution was adjusted to 0.1 % absorptivity.

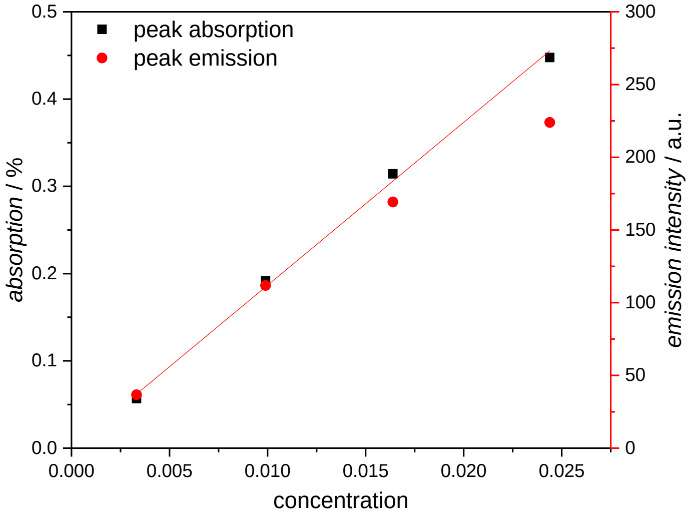

Figure S6: Comparison of peak absorption (470 nm) to peak emission (550 nm) intensity over concentration of **1** in water (*l* = 1 cm).

3.1.1. Fluorophores in H_2O

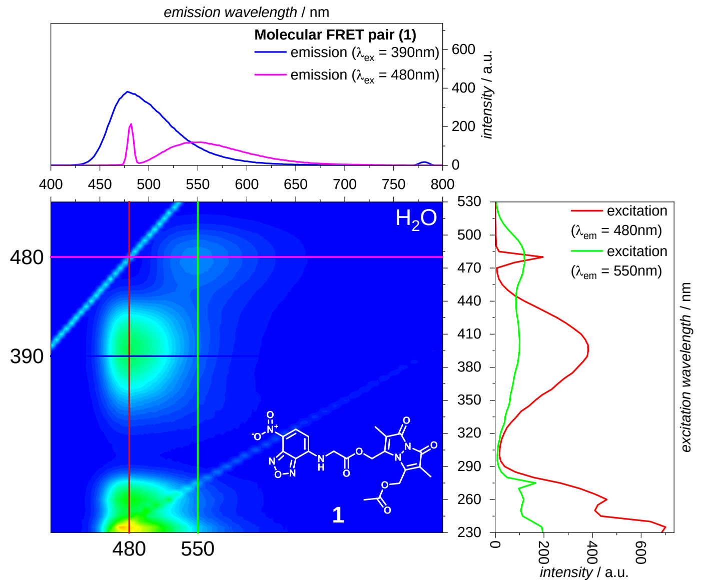

Figure S7: 3D Fluorescence spectrum of **1** in water with emission and excitation slices (*l* = 10 mm, sample concentration around 0.1 % absorption).

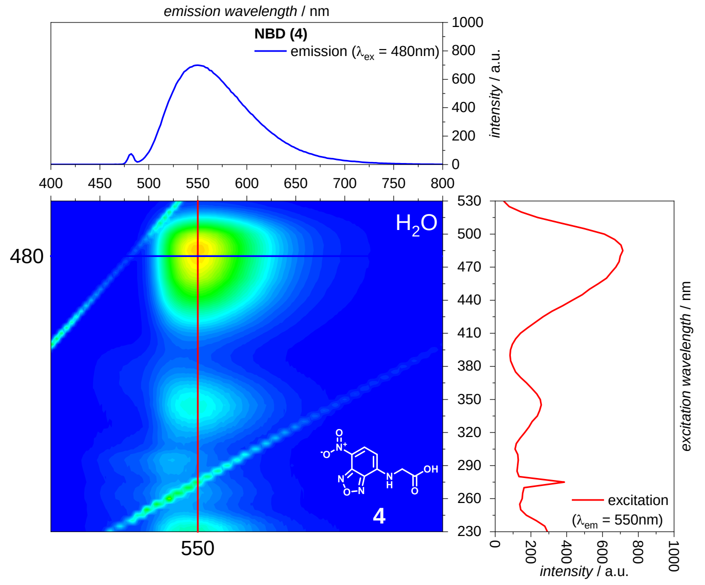

Figure S8: 3D Fluorescence spectrum of **4** in water with emission and excitation slices (*l* = 10 mm, sample concentration around 0.1 % absorption).

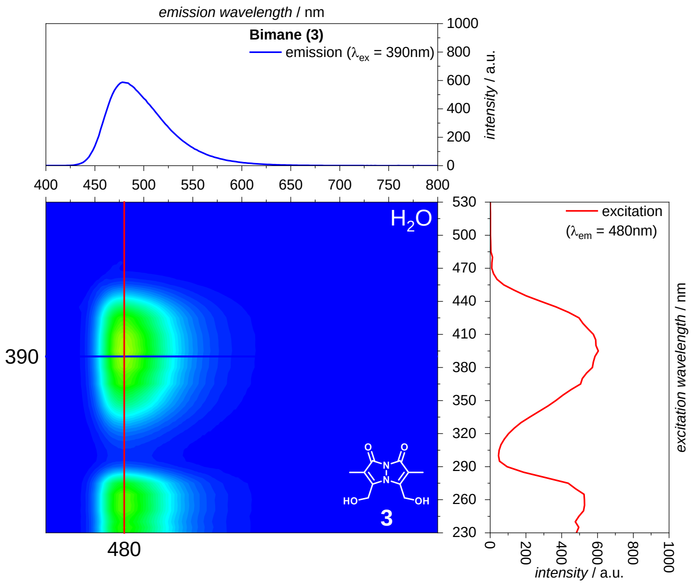

Figure S9: 3D Fluorescence spectrum of **3** in water with emission and excitation slices (*l* = 10 mm, sample concentration around 0.1 % absorption).

3.1.2. Fluorophores in THF

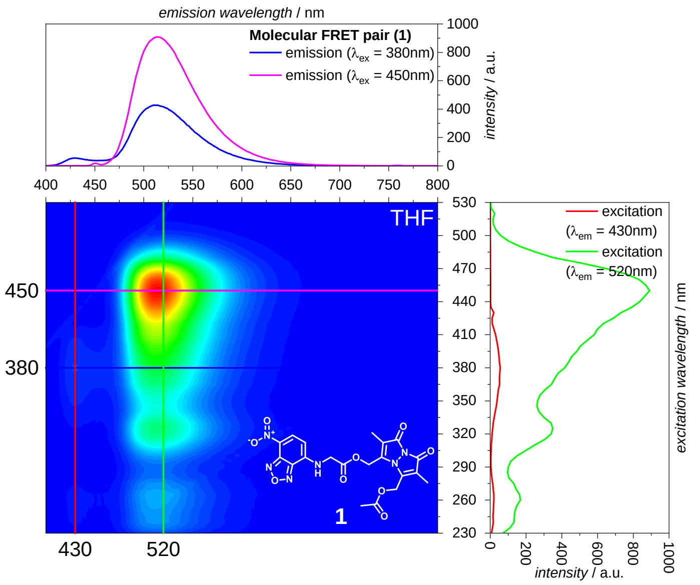

Figure S10: 3D Fluorescence spectrum of **1** in THF with emission and excitation slices (*l* = 10 mm, sample concentration around 0.1 % absorption).

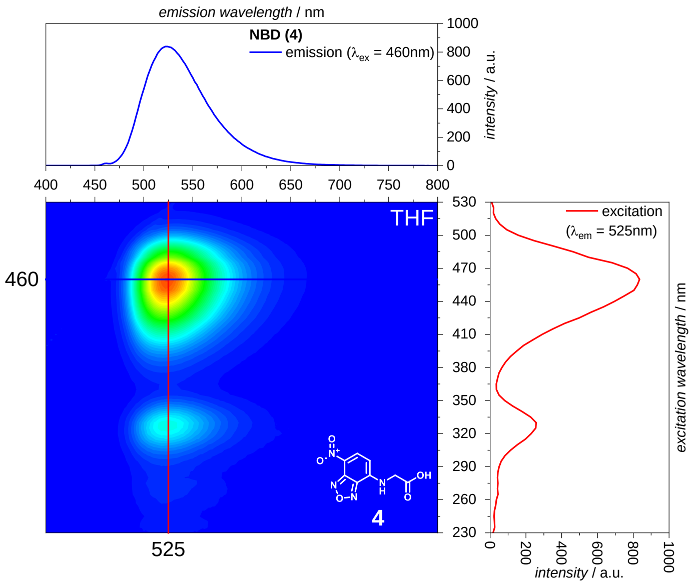

Figure S11: 3D Fluorescence spectrum of **4** in THF with emission and excitation slices (*l* = 10 mm, sample concentration around 0.1 % absorption).

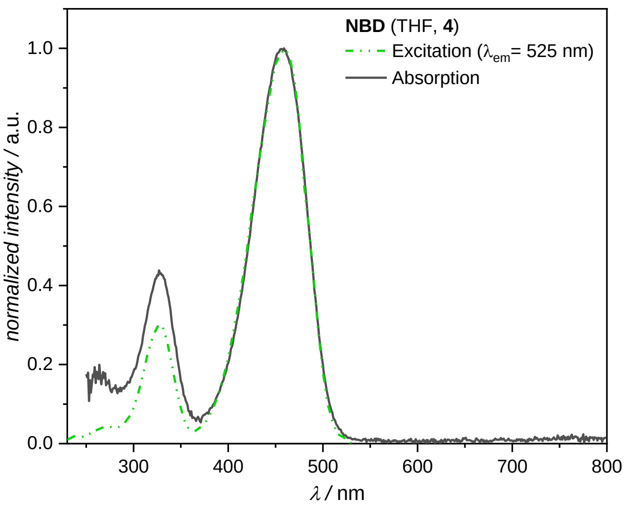

Figure S12: Overlap of the excitation spectrum (λ_{EM} = 525 nm) and the absorption spectrum of **4** in THF.

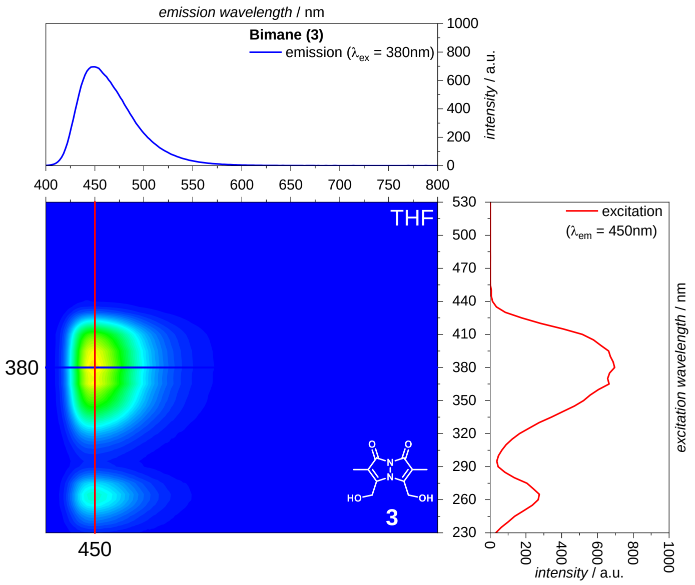

Figure S13: 3D Fluorescence spectrum of **3** in THF with emission and excitation slices (*l* = 10 mm, sample concentration around 0.1 % absorption).

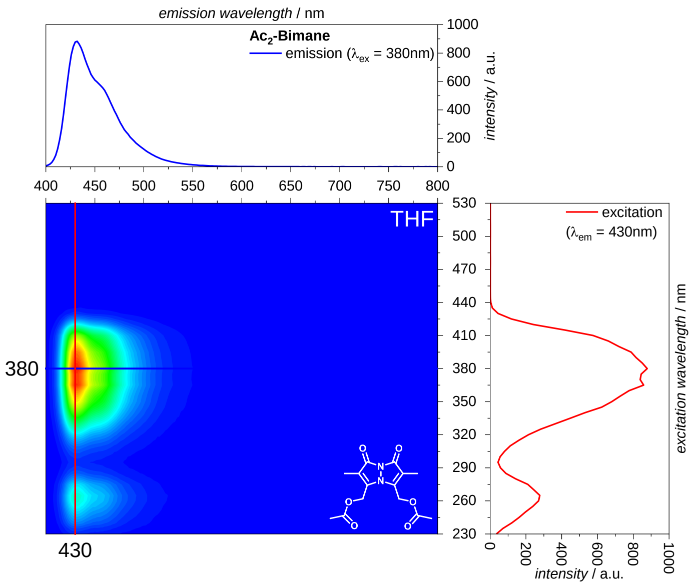

Figure S14: 3D Fluorescence spectrum of Ac_2-Bimane in THF with emission and excitation slices (*l* = 10 mm, sample concentration around 0.1 % absorption).

3.1.3. Irradiation Experiments

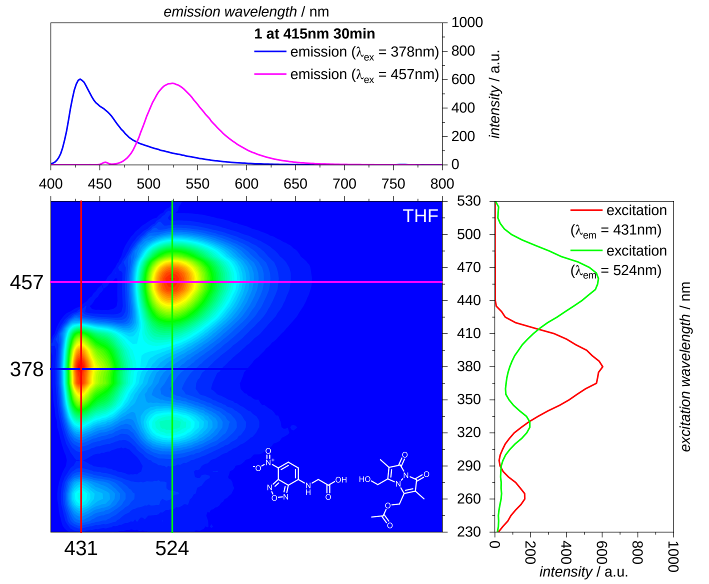

Figure S15: 3D Fluorescence spectrum of **1** irradiated with 415 nm for 30 min recorded in THF with emission and excitation slices (*l* = 10 mm, sample concentration around 0.1 % absorption).

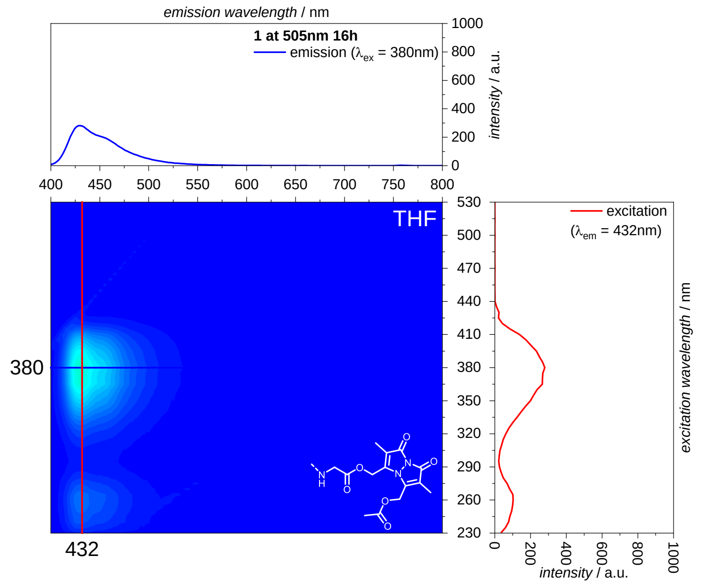

Figure S16: 3D Fluorescence spectrum of **1** irradiated with 505 nm for 16 h recorded in THF with emission and excitation slices (*l* = 10 mm, sample concentration around 0.1 % absorption).

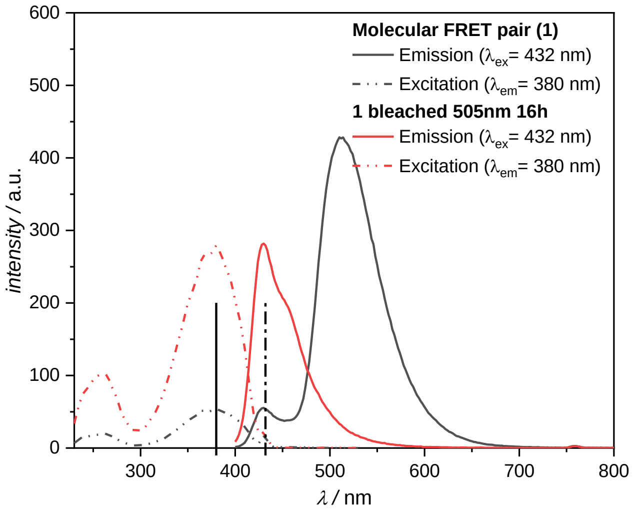

Figure S17: Fluorescence spectra of **1** before and after irradiating with 505 nm for 16 h recorded in THF (*l* = 10 mm, sample concentration around 0.1 % absorption, 3D experiment, PMT voltage = 570V).

3.1.4. Polymers

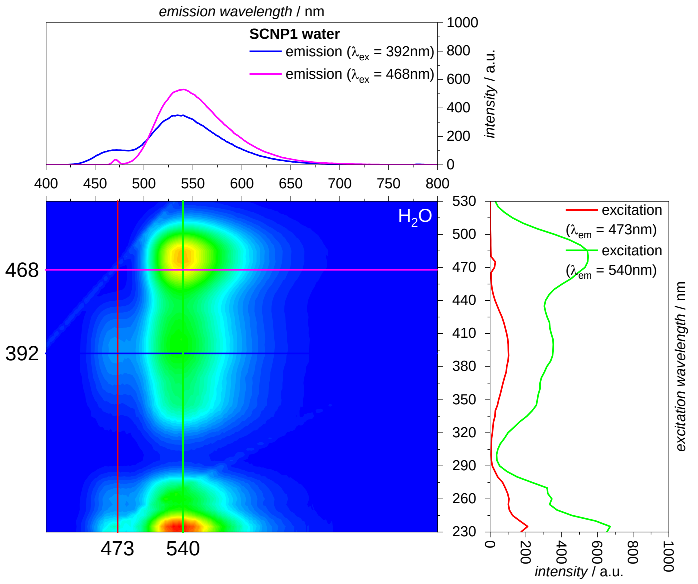

Figure S18: 3D Fluorescence spectrum of **SCNP1** in water with emission and excitation slices (*l* = 10 mm, sample concentration around 0.1 % absorption).

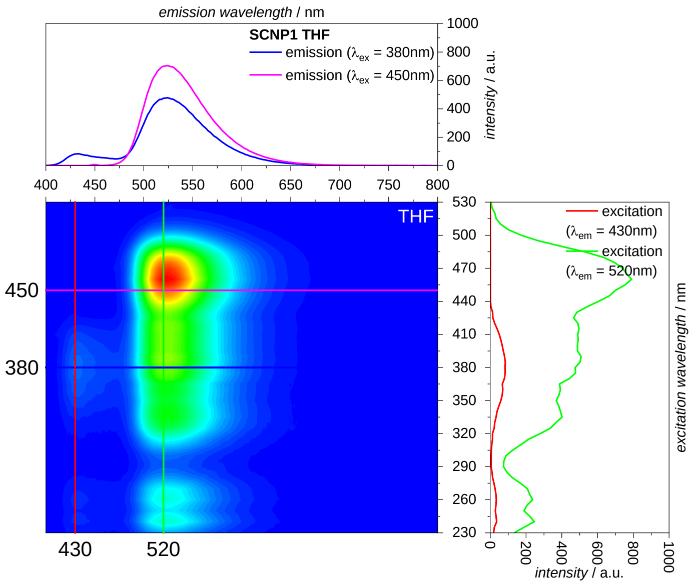

Figure S19: 3D Fluorescence spectrum of **SCNP1** in THF with emission and excitation slices (*l* = 10 mm, sample concentration around 0.1 % absorption).

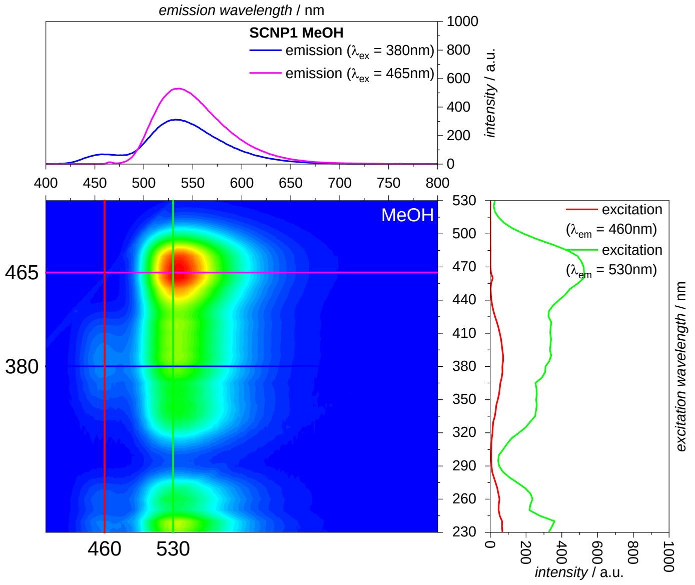

Figure S20: 3D Fluorescence spectrum of **SCNP1** in methanol (MeOH) with emission and excitation slices (*l* = 10 mm, sample concentration around 0.1 % absorption).

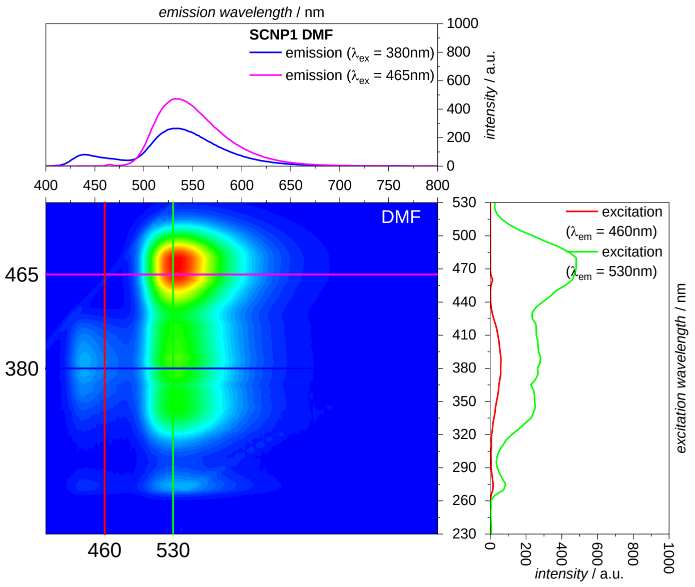

Figure S21: 3D Fluorescence spectrum of **SCNP1** in dimethylformamide (DMF) with emission and excitation slices (*l* = 10 mm, sample concentration around 0.1 % absorption).

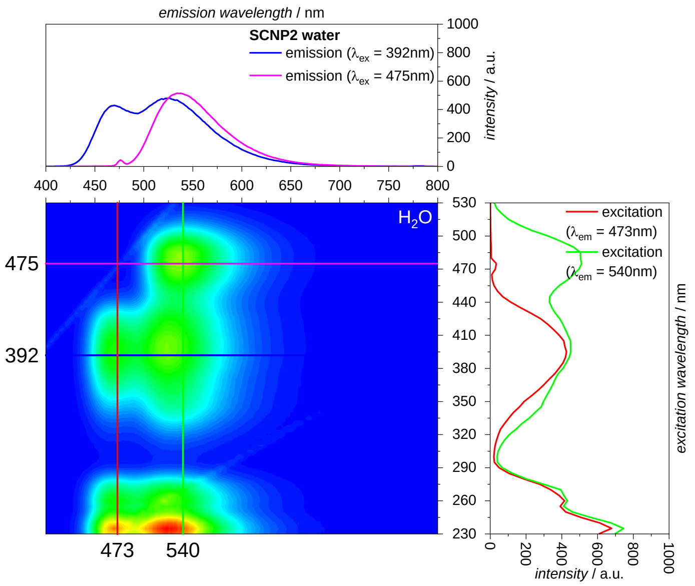

Figure S22: 3D Fluorescence spectrum of **SCNP2** in water with emission and excitation slices (*l* = 10 mm, sample concentration around 0.1 % absorption).

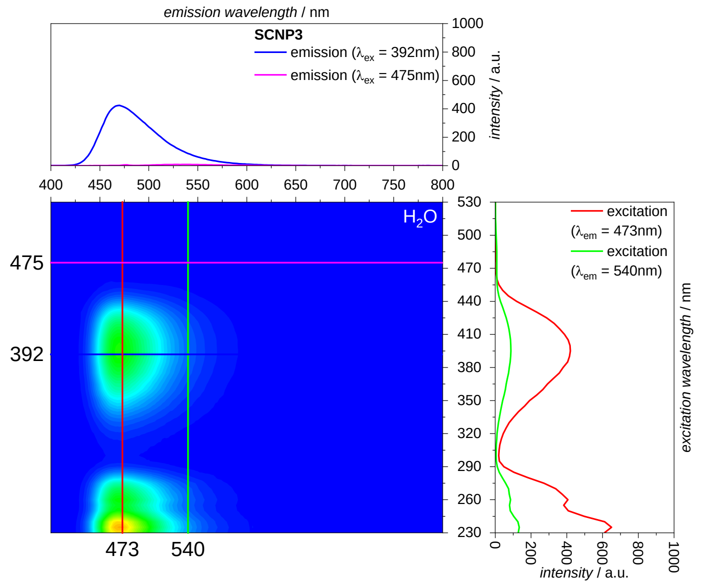

Figure S23: 3D Fluorescence spectrum of **SCNP3** in water with emission and excitation slices (*l* = 10 mm, sample concentration around 0.1 % absorption).

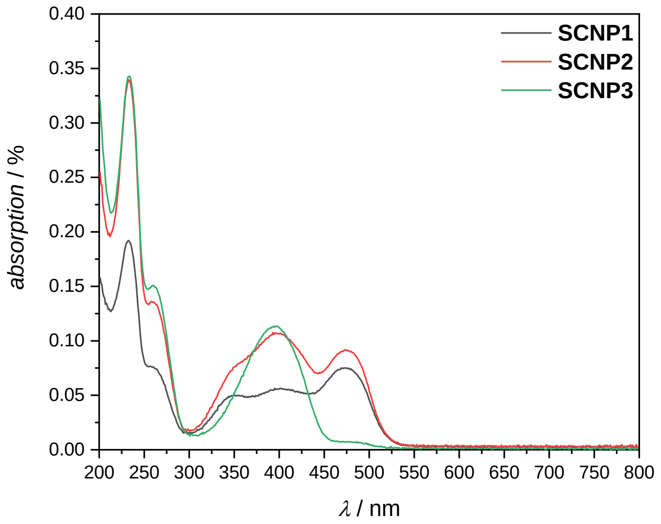

Figure S24: Absorption spectra of SCNP1-3 in water showing different absorptivity in the bimane (390 nm) and NBD (475 nm) region.

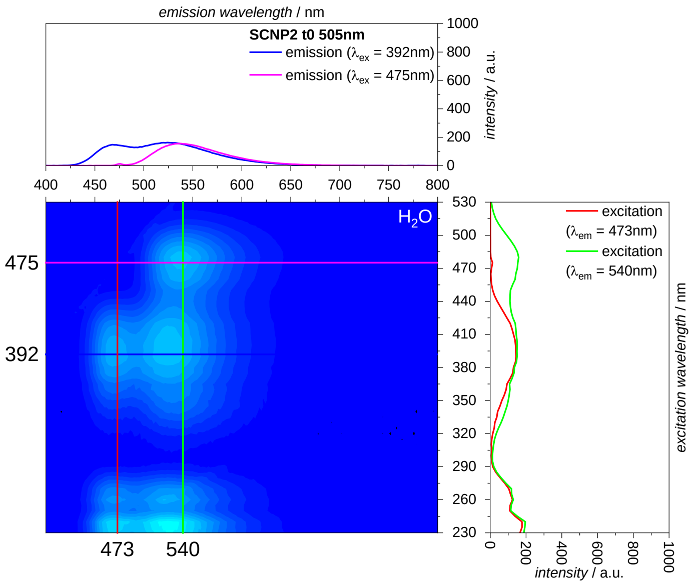

Figure S25: 3D Fluorescence spectrum of **SCNP2** before irradiating with 505 nm in water with emission and excitation slices (*l* = 10 mm, sample concentration around 0.1 % absorption).

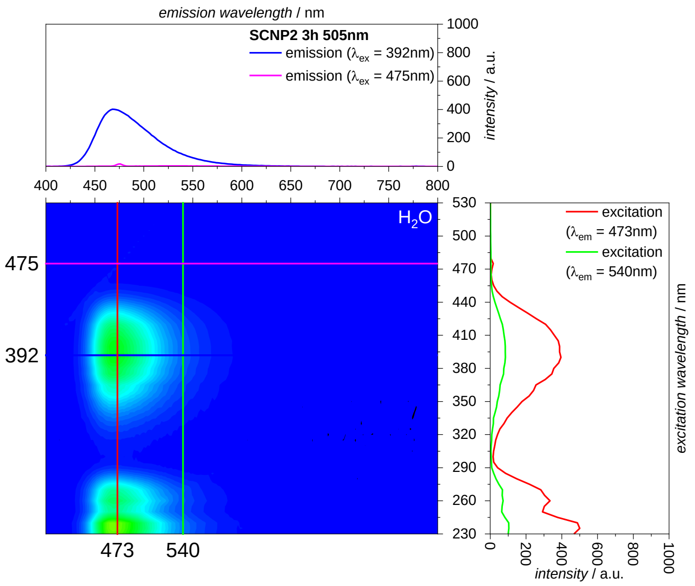

Figure S26: 3D Fluorescence spectrum of **SCNP2** after irradiating with 505 nm for 3 h in water with emission and excitation slices (*l* = 10 mm, sample concentration around 0.1 % absorption).

4. LCMS

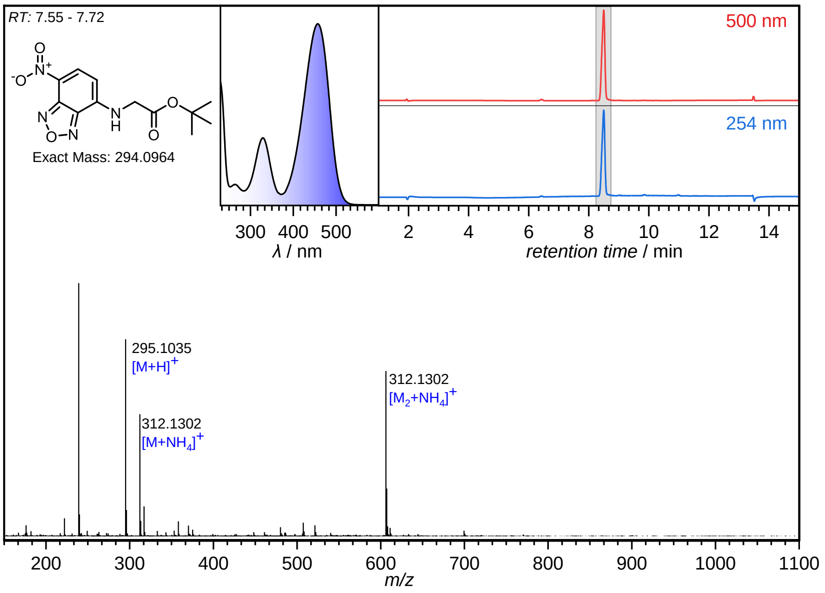

Figure S27: LC-MS trace (254 nm, 500 nm detector wavelength), absorption spectra and accumulated mass spectrum of *tert*-butyl (7-nitrobenzo[c][1,2,5]oxadiazol-4-yl)glycinate (NBD-Gly-*tert*).

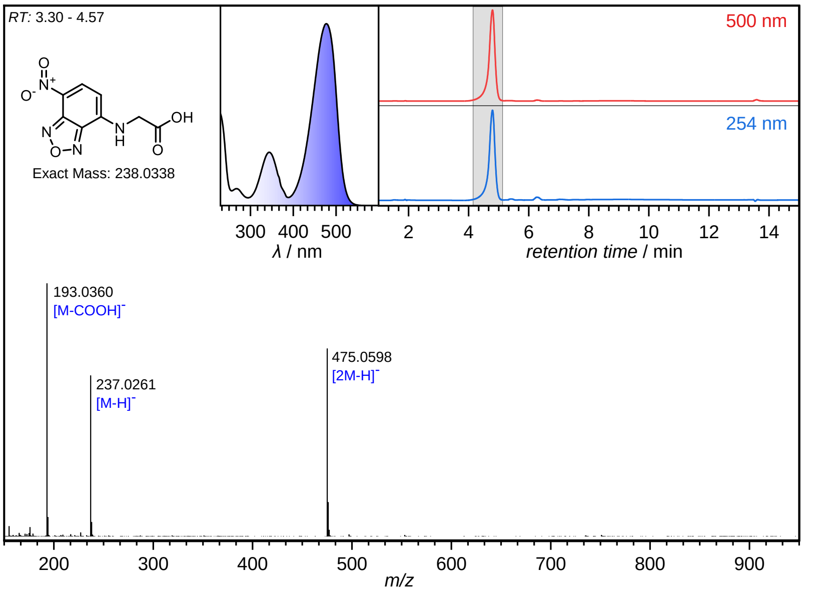

Figure S28: LC-MS trace (254 nm, 500 nm detector wavelength), absorption spectra and accumulated mass spectrum of **4**.

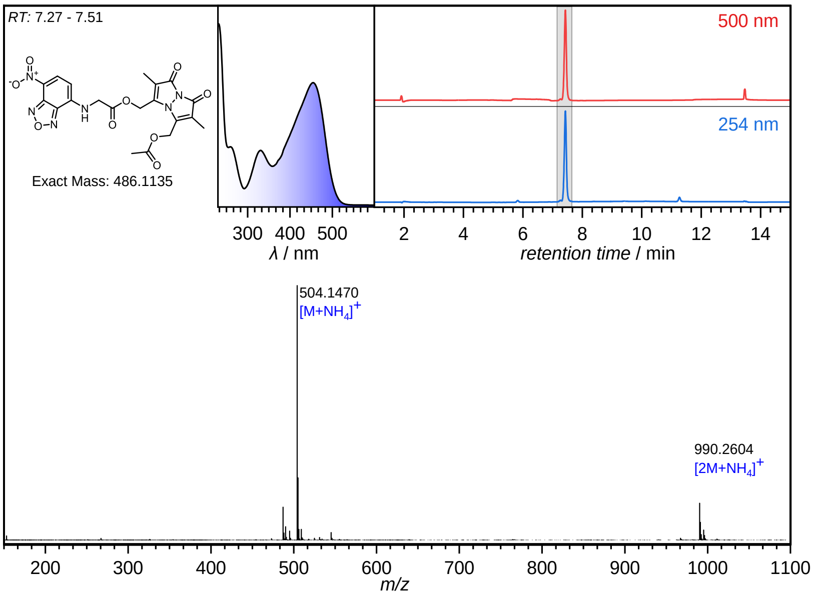

Figure S29: LC-MS trace (254 nm, 500 nm detector wavelength), absorption spectra and accumulated mass spectrum of **1**.

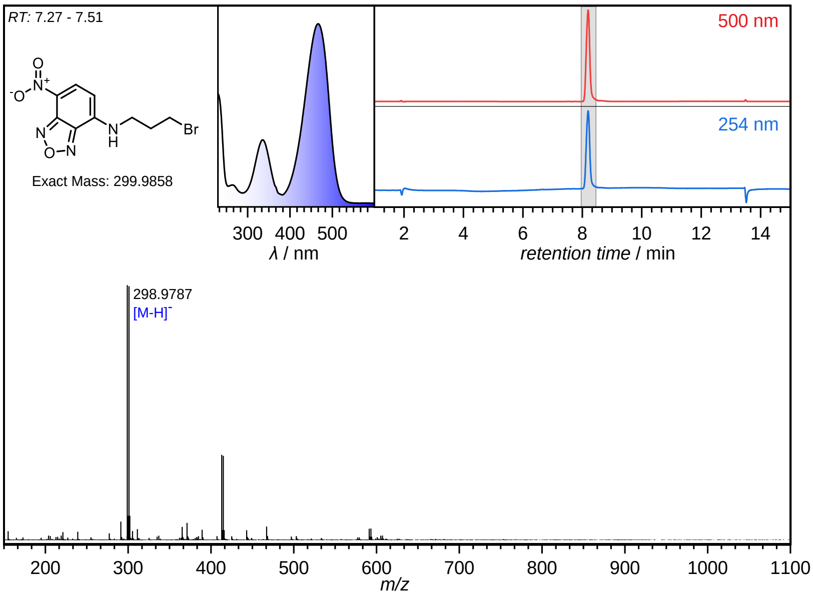

Figure S30: LC-MS trace (254 nm, 500 nm detector wavelength), absorption spectra and accumulated mass spectrum of **5**.

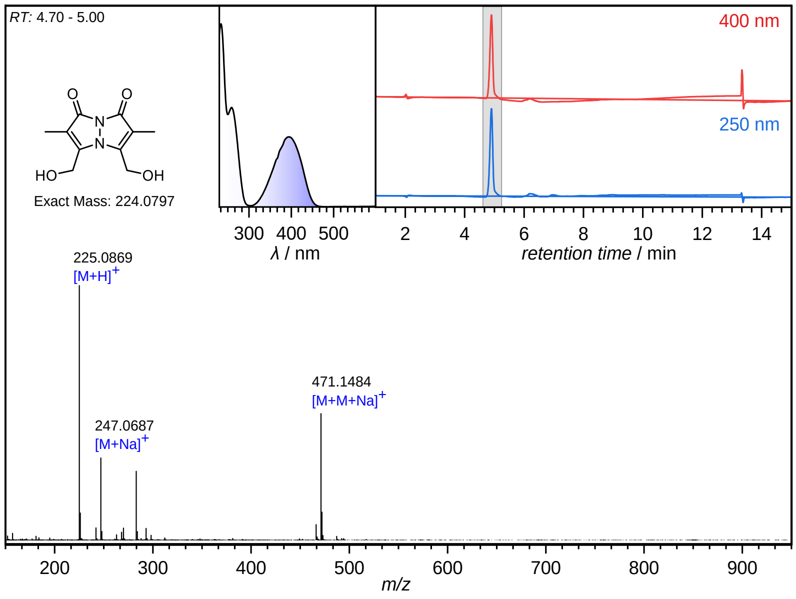

Figure S31: LC-MS trace (254 nm, 500 nm detector wavelength), absorption spectra and accumulated mass spectrum of **3**.

Table S2: Experimental and theoretical *m*/*z* of small molecule synthesis products**.**

Product	Symbol	m/z^{exp}	m/z^{theor}	m/z^{adduct}	Δ_{ppm}
NBD-Gly-*tert*	$[M+H]^+$	295.1035	294.0964	1.0073	0.61
**4**	$[M-H]^-$	237.0261	238.0338	1.0073	1.76
**1**	$[M+NH_4]^+$	504.1470	486.1135	18.0338	0.62
**5**	$[M-H]^-$	298.9787	299.9858	1.0073	0.60
**3**	$[M+H]^+$	225.0869	224.0797	1.0073	0.36

5. NMR Spectra

5.1. Polymer

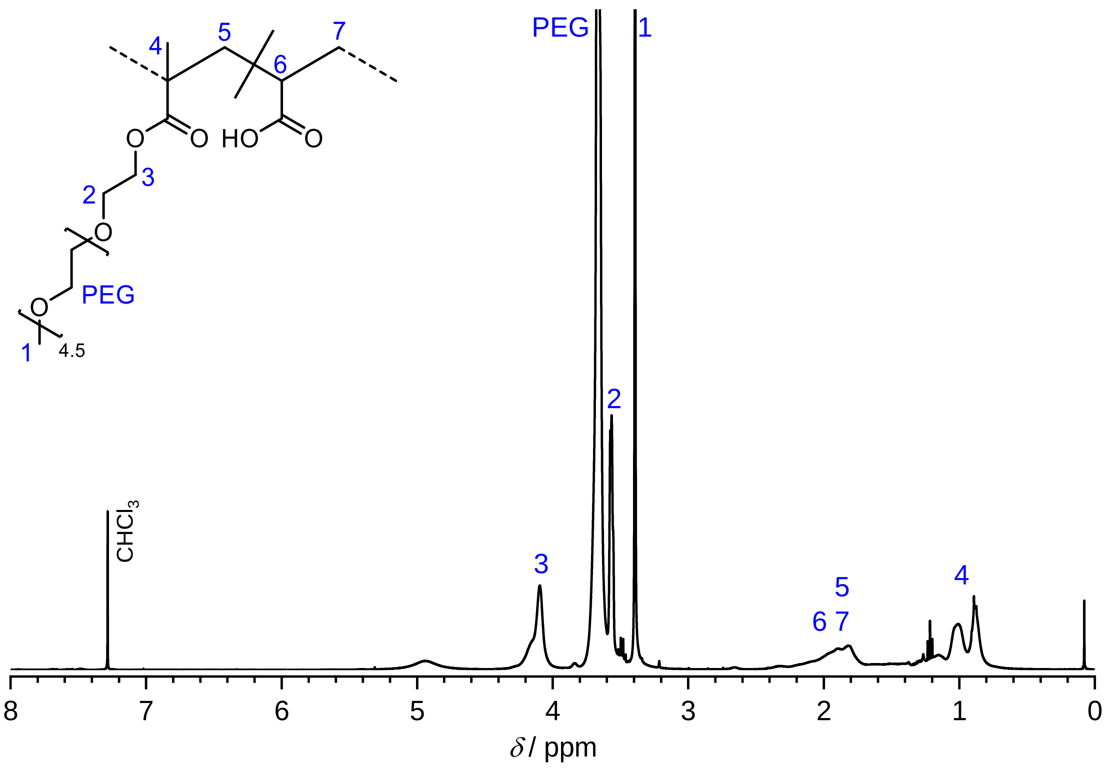

Figure S32: ^{1}H NMR spectrum of end group modification poly(MPEGMA-co-AA) recorded in $CDCl_3$ and assigned resonances (**P1**).

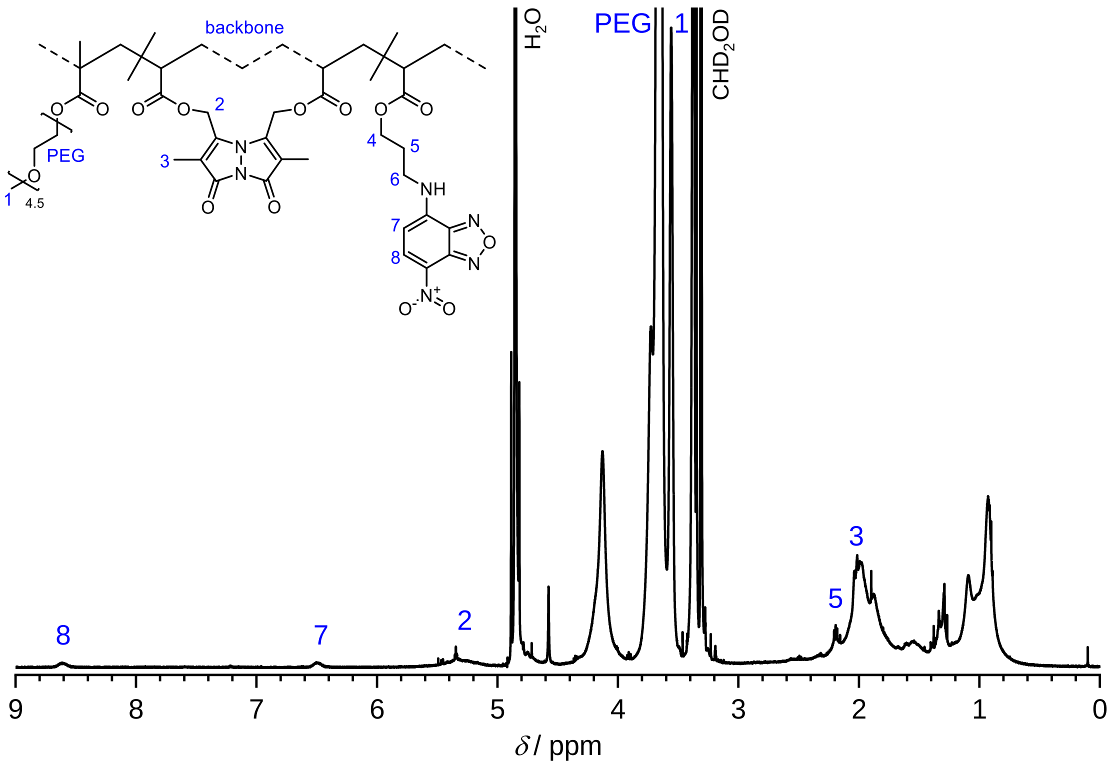

Figure S33: ^{1}H NMR spectrum of NBD post-functionalized and bimane cross-linked poly(MPEGMA-co-AA) recorded in CD_3OD and assigned resonances (**SCNP1**).

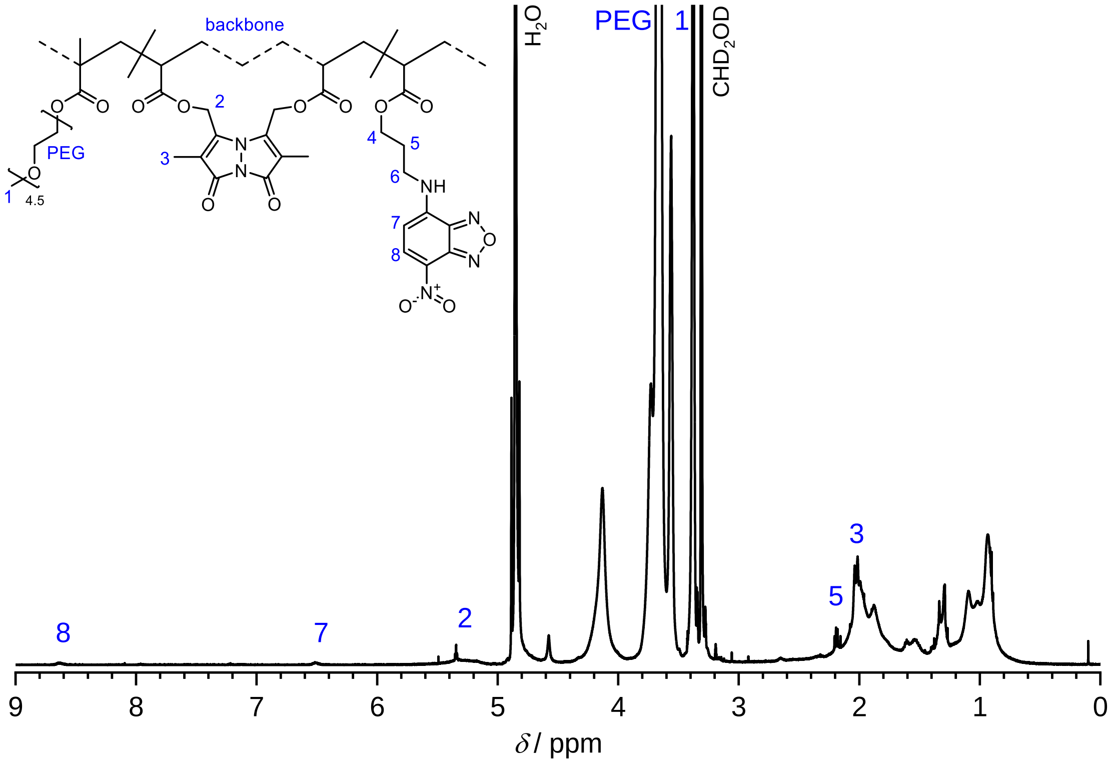

Figure S34: ^{1}H NMR spectrum of NBD post-functionalized and bimane cross-linked poly(MPEGMA-co-AA) recorded in CD_3OD and assigned resonances (**SCNP2**).

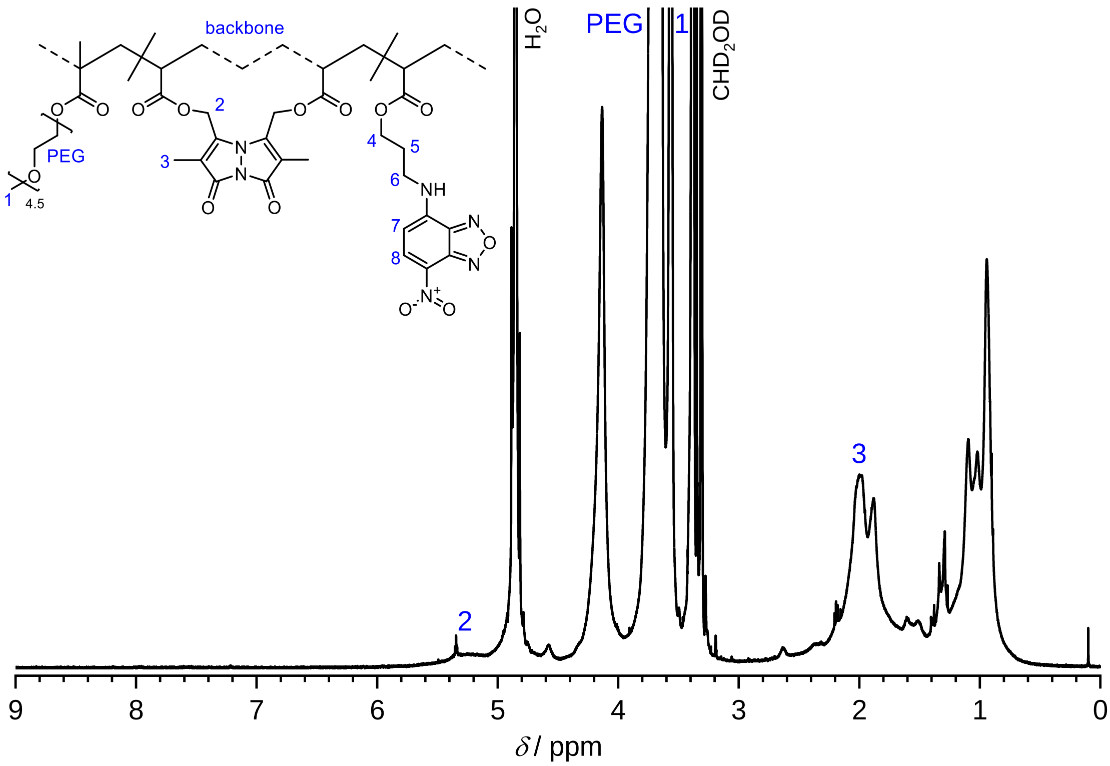

Figure S35: ^{1}H NMR spectrum of NBD post-functionalized and bimane cross-linked poly(MPEGMA-co-AA) recorded in CD_3OD and assigned resonances (**SCNP3**).

5.2. Small Molecule

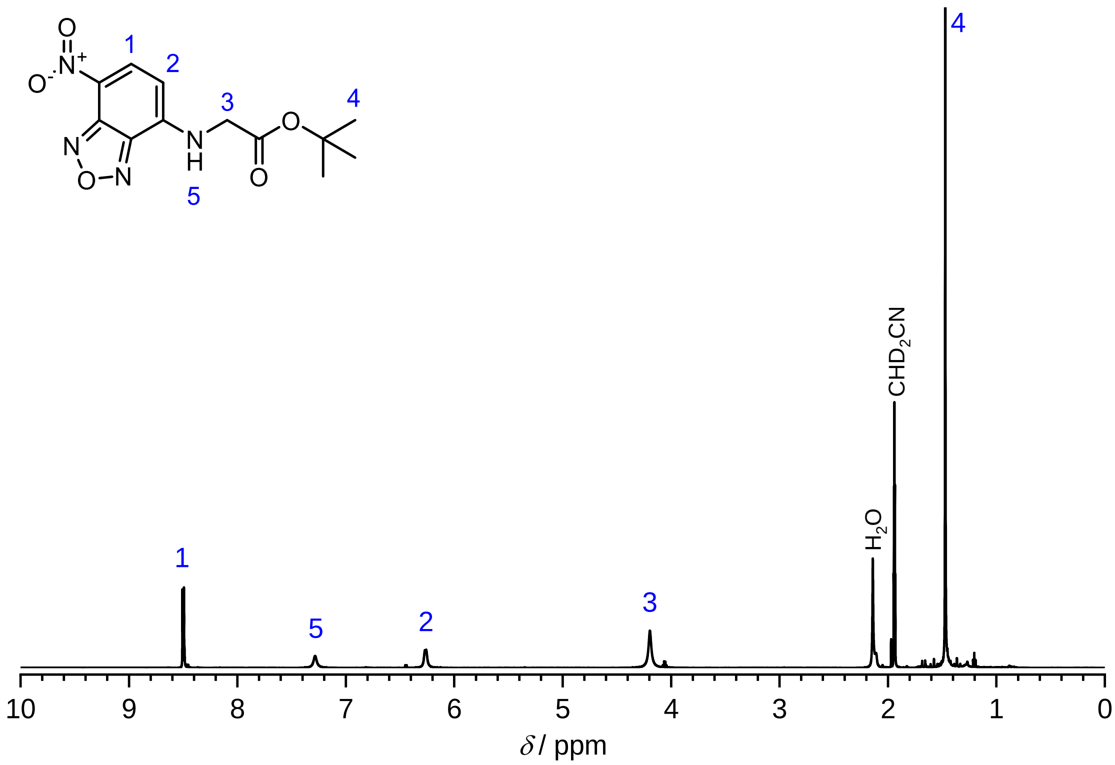

Figure S36: ^{1}H NMR spectrum of *tert*-butyl (7-nitrobenzo[c][1,2,5]oxadiazol-4-yl)glycinate (NBD-Gly-*tert*) recorded in CD_3CN and assigned resonances.

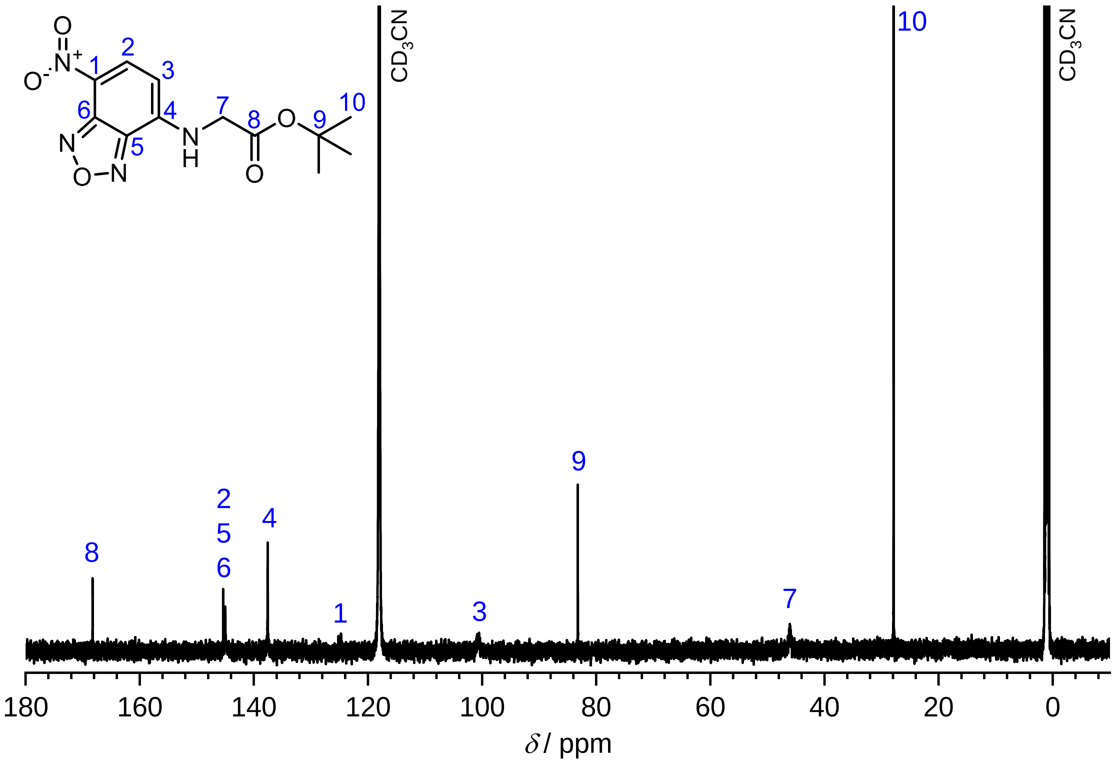

Figure S37: ^{13}C NMR spectrum of *tert*-butyl (7-nitrobenzo[c][1,2,5]oxadiazol-4-yl)glycinate (NBD-Gly-*tert*) recorded in CD_3CN and assigned resonances.

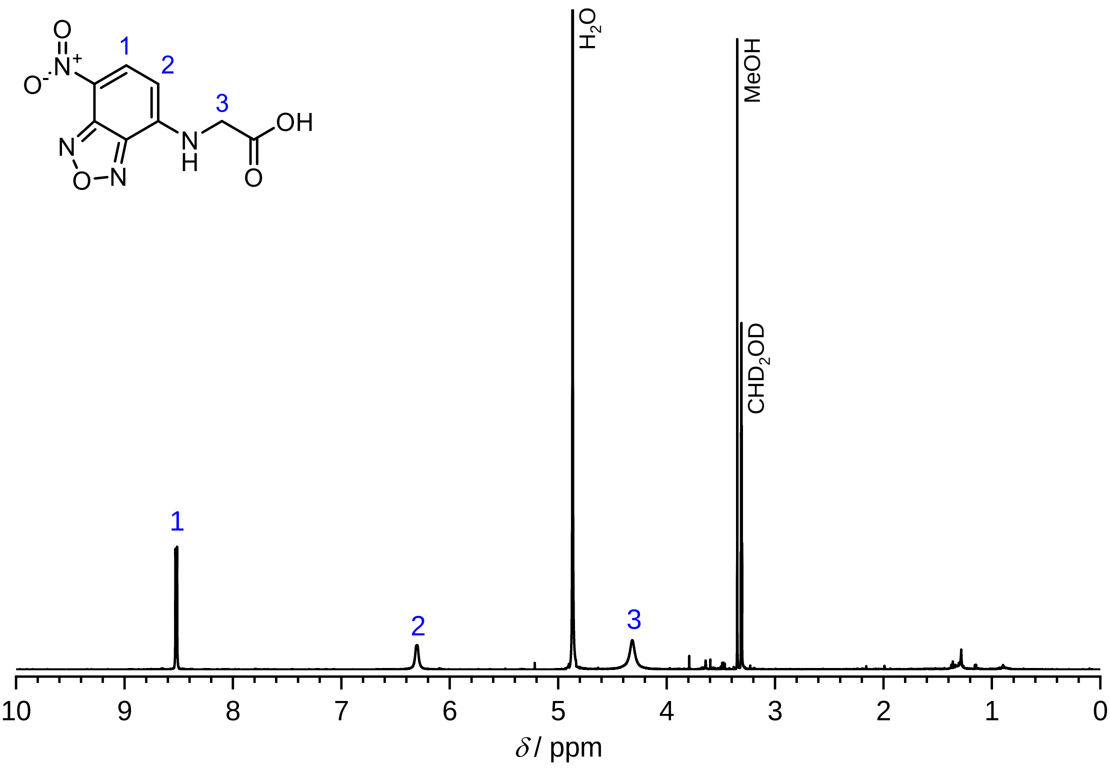

Figure S38: ^{1}H NMR spectrum of **4** recorded in CD_3OD and assigned resonances.

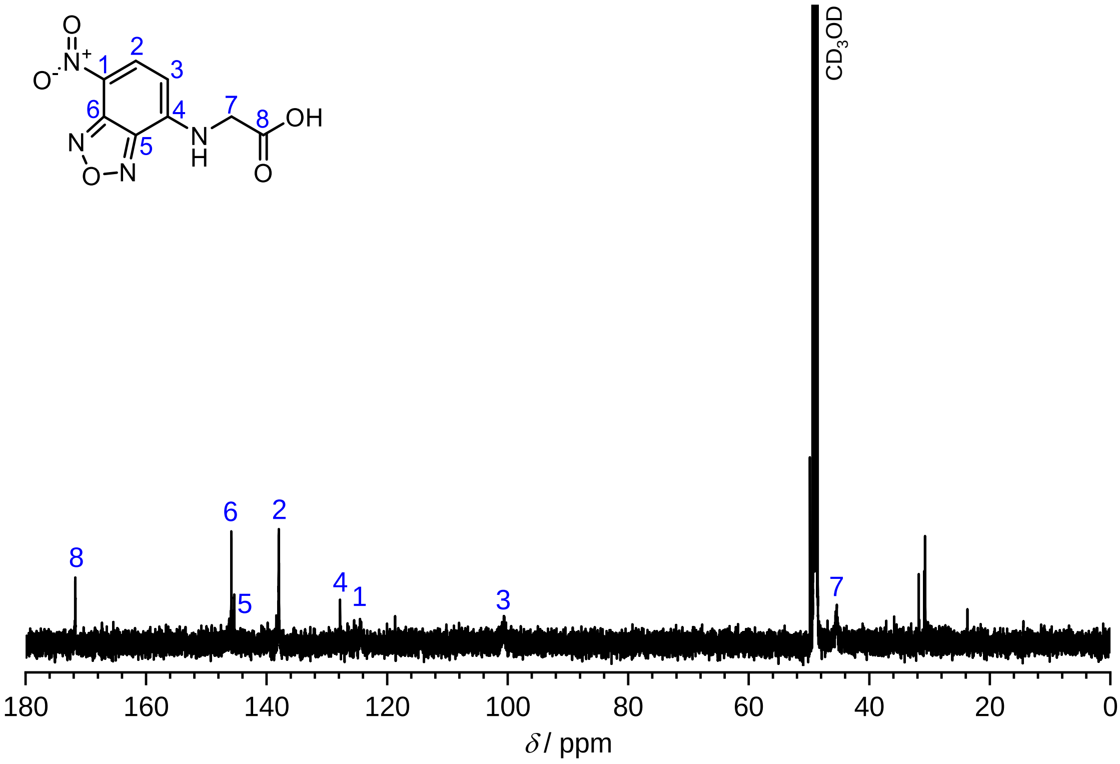

Figure S39: ^{13}C NMR spectrum of **4** recorded in CD_3OD and assigned resonances.

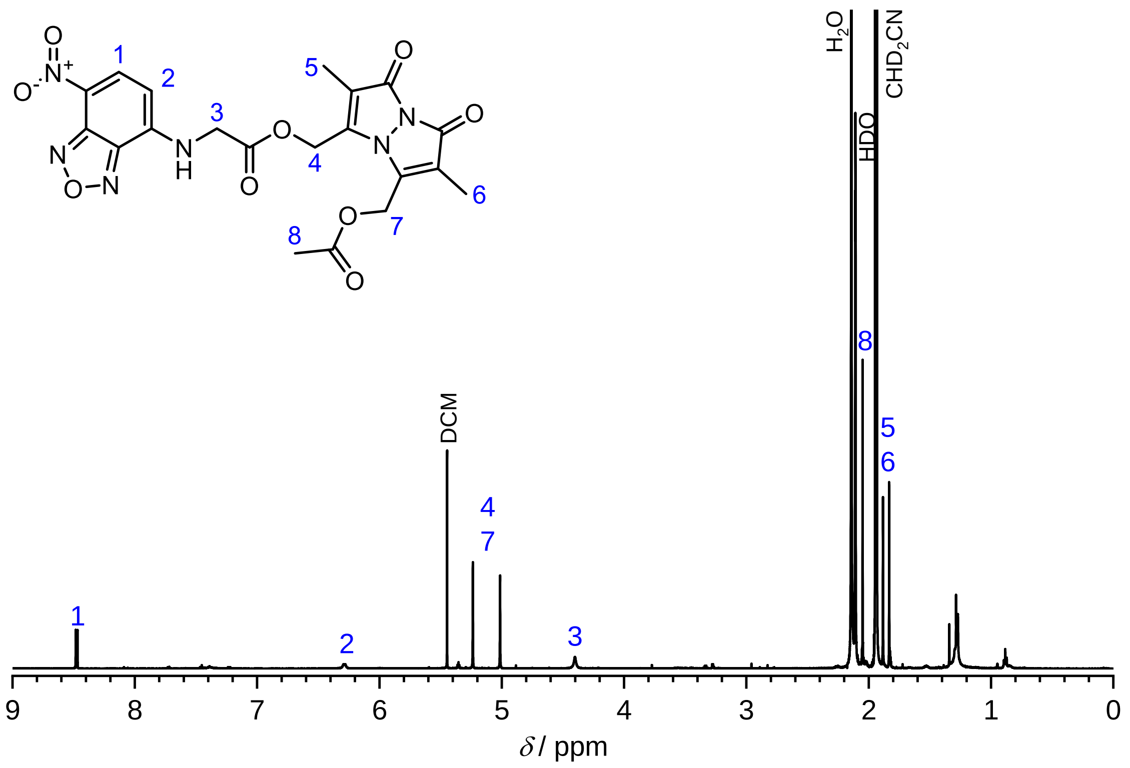

Figure S40: ^{1}H NMR spectrum of **1** recorded in CD_3CN and assigned resonances. The resonance at 1.29 ppm results from high boiling alkanes as part of impurity of cyclohexane and are difficult to avoid for small scale reactions. We assume that high boiling alkanes do not interfere with the photochemical reactions.

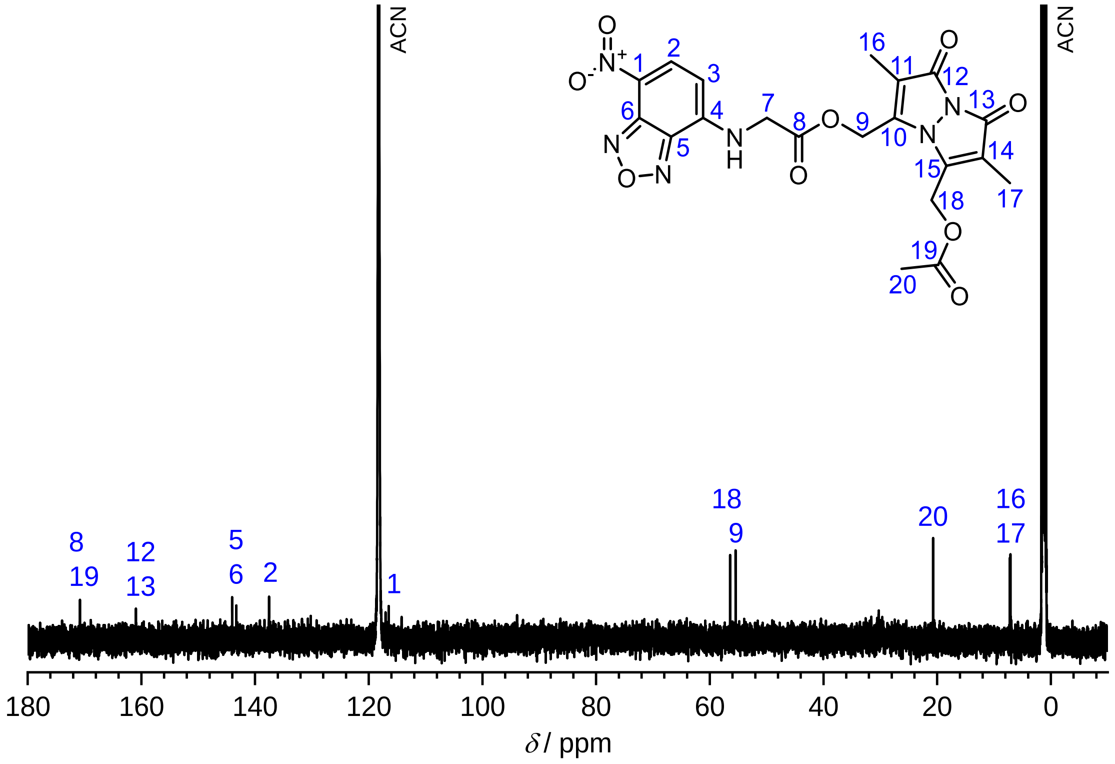

Figure S41: ^{13}C NMR spectrum of **1** recorded in CD_3CN and assigned resonances.

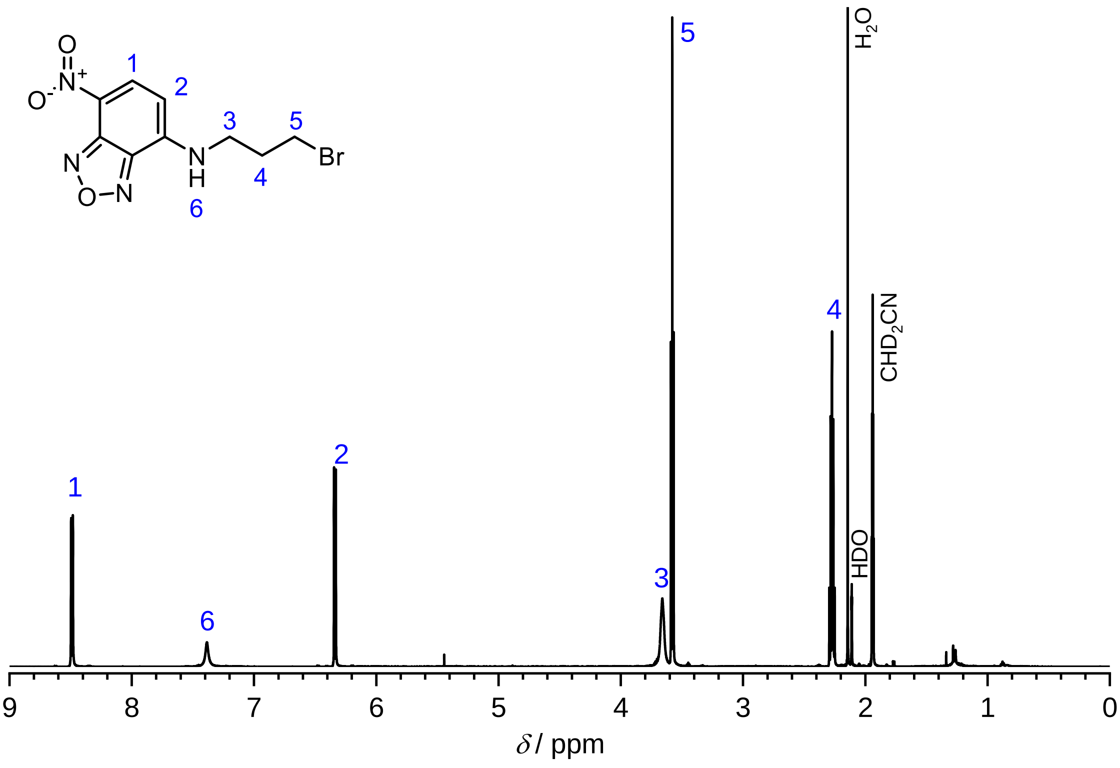

Figure S42: ^{1}H NMR spectrum of **5** recorded in CD_3CN and assigned resonances.

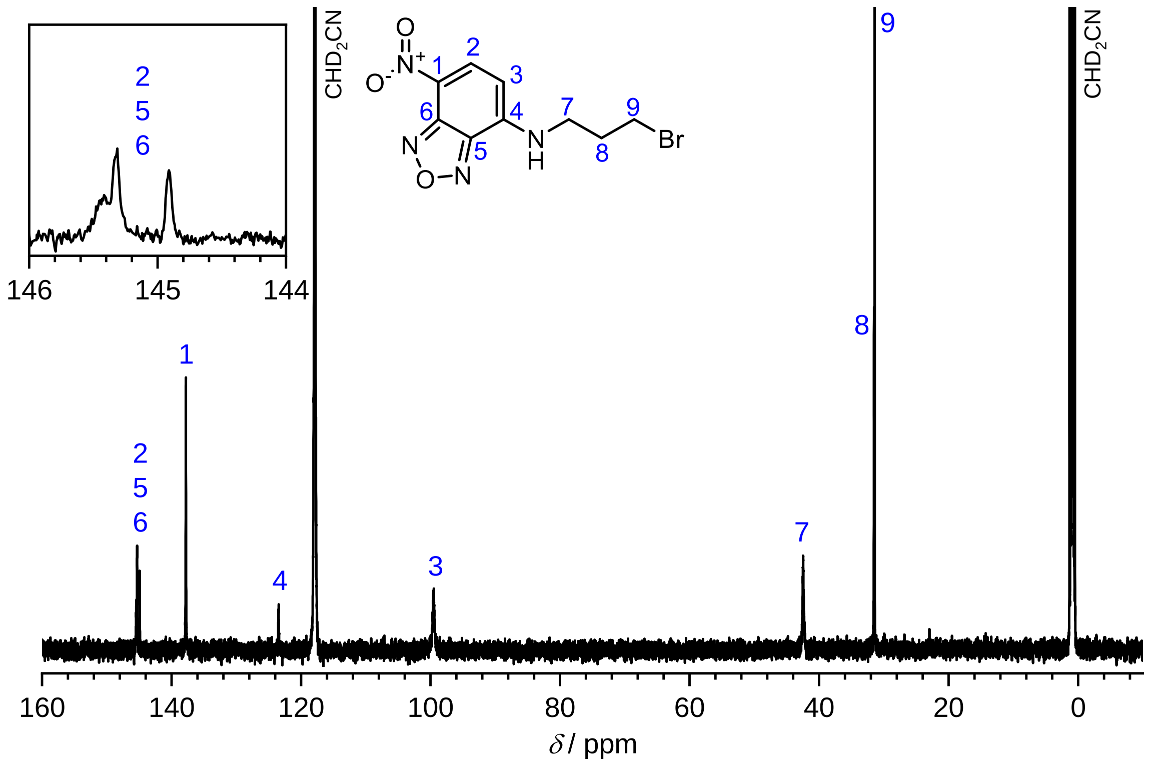

Figure S43: ^{13}C NMR spectrum of **5** recorded in CD_3CN and assigned resonances.

6. References

[1] G. R. Fulmer, A. J. Miller, N. H. Sherden, H. E. Gottlieb, A. Nudelman, B. M. Stoltz, J. E. Bercaw, K. I. Goldberg, *Organometallics* **2010**, *29*, 2176-2179.

[2] I. M. Irshadeen, V. X. Truong, H. Frisch, C. Barner-Kowollik, *Chem. Commun.* **2022**, *58*, 12975-12978.

[3] M. Dietrich, M. Glassner, T. Gruendling, C. Schmid, J. Falkenhagen, C. Barner-Kowollik, *Polym. Chem.* **2010**, *1*, 634-644.

[4] G. Greco, N. D’Antona, G. Gambera, G. Nicolosi, *Synlett* **2014**, *25*, 2111-2114.

[5] T. Buscher, Á. Barroso, C. Denz, A. Studer, *Polym. Chem.* **2015**, *6*, 4221-4229.
